# Bridging the Gap: Linking Molecular Simulations and Systemic Descriptions of Cellular Compartments

**DOI:** 10.1371/journal.pone.0014070

**Published:** 2010-11-22

**Authors:** Tihamér Geyer, Xavier Mol, Sarah Blaß, Volkhard Helms

**Affiliations:** Center for Bioinformatics, Saarland University, Saarbrücken, Germany; Auburn University, United States of America

## Abstract

Metabolic processes in biological cells are commonly either characterized at the level of individual enzymes and metabolites or at the network level. Often these two paradigms are considered as mutually exclusive because concepts from neither side are suited to describe the complete range of scales. Additionally, when modeling metabolic or regulatory cellular systems, often a large fraction of the required kinetic parameters are unknown. This even applies to such simple and extensively studied systems like the photosynthetic apparatus of purple bacteria. Using the chromatophore vesicles of *Rhodobacter sphaeroides* as a model system, we show that a consistent kinetic model emerges when fitting the dynamics of a molecular stochastic simulation to a set of time dependent experiments even though about two thirds of the kinetic parameters in this system are not known from experiment. Those kinetic parameters that were previously known all came out in the expected range. The simulation model was built from independent protein units composed of elementary reactions processing single metabolites. This pools-and-proteins approach naturally compiles the wealth of available molecular biological data into a systemic model and can easily be extended to describe other systems by adding new protein or nucleic acid types. The automated parameter optimization, performed with an evolutionary algorithm, reveals the sensitivity of the model to the value of each parameter and the relative importances of the experiments used. Such an analysis identifies the crucial system parameters and guides the setup of new experiments that would add most knowledge for a systemic understanding of cellular compartments. The successful combination of the molecular model and the systemic parametrization presented here on the example of the simple machinery for bacterial photosynthesis shows that it is actually possible to combine molecular and systemic modeling. This framework can now straightforwardly be applied to other currently less well characterized but biologically more relevant systems.

## Introduction

Modern computational systems biology aims at an overall description of the components, interactions, regulatory circuits, and metabolic fluxes in biological cells [1], [2]. The central challenge for such a systemic description is to set up a consistent network for the complete system [3]. To facilitate the generation of such large-scale models a number of databases have been set up which compile metabolic, regulatory, and genetic informations (e.g. KEGG, EcoCyc, Sabio-RK). At the other end of the spectrum are the molecular modeling approaches used in the fields of biochemistry and molecular biology which aim at understanding the functional details of individual proteins down to the atomistic level. Between these two paradigms there is a significant gap in scales which cannot easily be bridged from either side. Neither the existing network approaches nor the molecular modeling techniques can be applied to the full range of time and length scales from individual molecules to a complete compartment. Thus, there is a clear need for novel computational methods that have a resolution at the molecular level and propagate the system dynamics at the time scale of the biochemical reactions.

Here, we show that this gap between molecular and systems biology can be successfully bridged by combining our previously presented pools-and-proteins approach [4] with a systemic top-down parametrization of the set of individual kinetic and biophysical parameters against a set of time-dependent experimental data that probe the behavior of the full system. On the one hand, this allows for making full use of the vast amount of detailed biological knowledge about the molecular processes in and at the individual proteins for the setup of the computational model. On the other hand, the systemic treatment of the complete model enables a direct comparison between the, normally, macroscopic experiments and the behavior of the completely assembled system. In this stochastic model, a protein is an encapsulated object that undergoes individual microscopic reactions like the binding of a metabolite to its binding site, an electron transfer from a donor group to the active site, or the release of the product molecule back into the bulk. All these one-molecule-at-a-time reactions are modeled as stochastic events. At the next level, individual proteins are connected to metabolite pools. A metabolic model consequently consists of multiple independent copies of each type of protein and one pool per metabolite. Thus, the network is established without explicitly specifying pathways. All the details of the inner workings of the proteins are encapsulated locally so that the overall complexity remains at a manageable level. Due to the encapsulation the different protein types can even be modeled at different levels of internal details and individual proteins can be replaced by updated versions to incorporate new findings or amend shortcomings of the current model.

**Figure 1:**
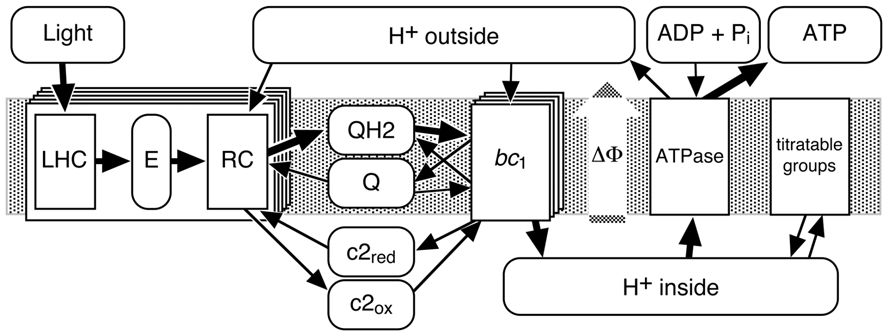
Network Representation of Bacterial Photosynthesis. The system consists of multiple copies of the proteins (rectangles) and one pool per metabolite (rounded rectangles). The thick arrows denote the flow of the energy through the system, i.e., its conversion from light energy via the intermediate forms of electron-proton pairs on the QH2 or as protons pumped into the vesicle into chemical energy stored on the ATP molecules. The grey area indicates the membrane with the cytoplasm above and the inside of the vesicle below.

To demonstrate the power of such a bottom-up modeling approach combined with a systemic parameter determination, we used the simple and well understood photosynthetic apparatus of the purple bacterium *Rhodobacter* (*Rb.*) *sphaeroides* and compared the dynamic behavior of a molecular-stochastic model to a set of time-dependent experiments. The selected experiments were taken from a project which investigated the role of the PufX protein in cyclic electron transfer. They were conducted in the group of Oesterhelt and published in two consecutive papers [5], [6]. The versatile *Rb. sphaeroides*, which can live on respiration, fermentation, or photosynthesis, is one of the best studied bacteria [7], [8]. Its photosynthetic apparatus is located in the inner membrane of the bacteria and on so called chromatophore vesicles. The photosynthetic apparatus consists of four integral membrane proteins, the light-harvesting complexes (LHC) LH1 and LH2, the photosynthetic reaction center (RC), the proton pumping cytochrome *bc*_1_ complex, and the F_O_-F_1_-ATP synthase (ATPase). It also contains the two electron carriers cytochrome *c*_2_ and ubiquinone (Q). The chromatophore vesicles have an average diameter of 45 to 60 nm only [9] so that the total number of membrane proteins per vesicle is less than a hundred, most of which are the simple LHCs [10], [11]. All relevant reactions take place inside the vesicle, which yields well defined boundary conditions for a numerical simulation of manageable complexity. Although these vesicles may also contain a few copies of the cytochrome *c* oxidase from the respiratory chain, experimental studies of the photosynthetic apparatus typically poison these proteins by adding potassium cyanide, so that there is no interference with this competing metabolic pathway [5]. Also, most of the other proteins embedded in the inner membrane of bacteria might be found in the membrane of a chromatophore vesicle, too. However, as photosynthesis works well in the absence and in the presence of these proteins, we did not consider them explicitly in our model. During photosynthesis, light energy is converted into chemical energy, which is then used to power the metabolism of the bacteria. The processes of this conversion are sketched in figure 1. First, photons are absorbed by the bacteriochlorophylls of the LHCs. Their energy is used in the RCs to translocate an electron from the special pair bacteriochlorophylls (P) to a quinone bound to the RC at the Q_b_ site. The charge of the electron on the Q is compensated by a proton taken up from the cytoplasm. Loaded with two such electron-proton pairs, the resulting quinol (QH2) unbinds and diffuses inside the membrane to the cytochrome *bc*_1_ complex. There, the protons are released into the vesicle interior and the energy of the two electrons is used in the so called Q-cycle [12] to pump another two protons into the vesicle. The resulting proton gradient, which leads to a transmembrane potential gradient ΔΦ, is finally used by the ATPase to produce ATP from ADP and inorganic phosphate.

Although bacterial photosynthesis is usually considered well understood, the available descriptions are rather qualitative representations similar to figure 1, whereas a quantitative computer simulation requires “hard” numbers for protein copy numbers and rate constants. Also, spatial configurations which put constraints on the kinetics have to be identified and their effect has to be quantified. As a first step we previously set up a steady state model of a chromatophore, for which we determined the stoichiometries of the membrane proteins and of the electron carriers cytochrome *c*_2_ and ubiquinone by using experimentally determined stoichiometries and spatial constraints such as the surface area of the vesicle [10], [13]. We found that a typical chromatophore vesicle from *Rb. sphaeroides* of 45 nm diameter contains about ten dimeric core complexes of two RCs and a Z-shaped LH1 each [10], [11], [14]. Most of the remaining surface area is occupied by the auxiliary LH2 with about six LH2 per core complex. All-atom molecular dynamics simulations of LHCs and core complexes in membranes support the hypothesis that the bent core complexes induce the formation of the chromatophore vesicles [15], [16]. Dynamic experiments showed that each vesicle contains on average a single ATPase [9]. Interestingly, the number of cytochrome *bc*_1_ complexes could only be determined by us with a rather large uncertainty. Many other kinetic parameters were left unconstrained, too, by this steady state reconstruction. A systems biological steady state reconstruction based on elementary modes was recently presented by Klamt et al [17].

As light serves as the central in-flux “metabolite”, the photosynthetic apparatus can be probed very easily in a wide range of kinetic regimes. These range from the picosecond time-scale for electronic excitations in the LHCs and RCs probed by single flash experiments over association and dissociation reactions on the millisecond range up to quasi steady state conditions under constant illumination. We previously presented a first model how the encapsulated objects representing the individual proteins are built up from their microscopic reactions [4]. In the following section we shortly summarize the implementation and explain how the cytochrome *bc*_1_ complex was extended from its original implementation in order to include more biological detail required to extract quantities that were measured in the experiments. The main focus of this publication is placed on the systemic parametrization of the underlying molecular stochastic model. By fitting against experiments covering all these time regimes [5], [6] we were able in this work to identify those reactions which are important for the dynamic behavior of the photosynthetic apparatus and to determine their rate constants. Interestingly, the experiments used as basis measured only four different observables. However, due to the wealth of information contained in the different time series even those reactions that are “far away” in the network from the measured quantities could be parametrized. Consequently, the simple and well understood photosynthetic apparatus of *Rb. sphaeroides* turned out to be an ideal system to develop and test our approach, which now allows to straightforwardly bridge between the molecular and the systemic realms of computational biology for other systems of more current interest, too.

However, the model of the bacterial photosynthesic apparatus presented here is far from complete. It is focussed on the metabolic processes taking place on the millisecond to second timescales, whereas any regulatory adaptations to slowly changing environmental conditions are omitted. On the other hand, the very fast multi-step exciton and electron dynamics in the LHCs are described by a few low-detail effective reactions in the current iteration of the model. This simplified picture was sufficient because these processes were not resolved in the experiments. These two examples show that the model of bacterial photosynthesis as presented here serves well as a versatile scaffold or template that can be used to collect knowledge about the individual proteins and about how they interact to reproduce the rich spectrum of experimental observations. It nevertheless proofs that this concept can be used to bridge between the two currently not so well connected fields of molecular and systems biology.

## Results and Discussion

In this work we report on how we connected a molecular model of a biological system to its systemic treatment to bridge between these two fields. This two-sided approach is reflected in the following section, too, where we first explain the molecular modeling aspects required for the stochastic simulations, which serve as the starting point from the one side of the gap, and then how we performed the optimization by treating the complete model as one entity. Each of the tasks could be performed independently but by combining them we are able to connect the molecular and the systemic sides by exploiting the respective strenghts of both approaches. After the model setup we show how different numbers of cytochrome *bc*_1_ complexes affect the non-equilibrium dynamics of the chromatophore vesicles. This initial analysis is then followed by the main part of this work, the stochastic dynamics simulations combined with an evolutionary parameter optimization of the full vesicles.

### The Chromatophore Model

#### Molecular Stochastic Simulations

The stochastic simulations of the chromatophore vesicles from *Rhodobacter sphaeroides* presented in this work were performed with a refined version of the model introduced in [4]. In the spirit of a bottom-up approach, the model vesicle contains multiple, independent copies of all protein types. Each protein copy is assembled from elementary microscopic reactions such as the transfer of a single electron, the binding of a metabolite molecule to a binding site of the protein, or the translocation of individual protons. By nature, each of these single-molecule reactions has to be simulated stochastically. In contrast to other approaches based on stoichiometric matrices (flux balance analysis [18], extreme pathways [19], elementary mode analysis [20]), or on propagating dynamic rate equations (for an introduction see [21]) which consider one reaction per protein type regardless of how many copies of the proteins are present, our model vesicle consists of as many independent copies of each of the proteins as are located in a real chromatophore vesicle. Consequently, our model also differs from the stochastic Gillespie approach for well stirred chemical systems [22] where the events in different copies of a protein are coupled to each other.

In our model, the proteins are connected to each other via metabolite pools which are well-mixed subvolumes of the simulated system. Hereby, different charge or oxidation states of the same biological molecule are treated as different metabolites. For example, the oxidized and reduced forms of cytochrome *c*_2_ are two distinct species. As the chromatophore vesicles are so small that the diffusive transport through the vesicle is much faster than the respective times for association and dissociation [13], a single compartment per metabolite is sufficient in the vesicle interior or in the membrane. Due to this distinction between the active proteins and the passive metabolite pools, we termed this simulation scheme the “pools-and-proteins” approach [4].

The initial conditions of a simulation are set via the numbers of metabolites in the pools. In a dark-adapted vesicle all cytochrome *c*_2_ are reduced. Consequently, all *c*_2_s will be in the pool representing the reduced cytochrome *c_2_* while the pool of oxidized *c_2_* is empty. At the same time, about 90% of the quinones will be in the QH2 pool. Before the actual experiments were simulated by “exposing” the model vesicle to the respective illumination profiles, the simulations were “thermalized” for 20 to 100 milliseconds in the dark.

The state vector of the simulation is propagated by looping over all individual reactions of the individual proteins and checking whether the conditions that allow a certain reaction to take place are fulfilled. A binding reaction, e.g., may only take place if the binding site is empty, and an electron transfer can only occur when the donor is reduced and the acceptor oxidized. If the conditions are fulfilled, the probability that the reaction takes place during the next time step of size Δ*t* is determined. The probability *P*_on_ for an association reaction at a certain binding site of an individual protein during Δ*t* depends on the concentration *ρ* of the metabolite in the pool and the association rate *k*_on_ as
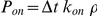
For dissociation or internal charge transfer reactions, which are independent of the metabolite concentrations, a survival time *τ*_off_ is determined following Gillespie [22] from the rate *k*_off_ and an exponentially distributed random number *r*:
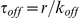
Based on τ_off_ a timer is initialized which then initiates the actual dissociation event. Note that such a waiting time algorithm cannot be used for the density dependent associations without extensive bookkeeping, because the metabolite concentrations may change after the timer was initialized due to other reactions that produce or consume the same metabolites.

In our approach none of the microscopic reactions was implemented as reversible. Explicitly reversible processes such as the association/dissociation of a metabolite or the back-and-forth hinge motion of the Rieske domain of the cytochrome *bc*_1_ complex were modeled as two independent elementary reactions each with their own conditions and their own rate constants. For the regimes considered here thermodynamically driven reverse reactions can be neglected.

As a consequence of our implementation from individual microscopic reactions we do not have to deal with the combinatorial explosion of the number of states which appears in traditional rate-equation approaches from treating all possible combinations of charge and oxidation states of the multiple sites in the proteins. A simple protein unit such as the reaction center can be modeled with two external binding sites for cytochrome *c*_2_ and quinone, two sites for electrons residing on the special pair and the Q_b_ quinone, respectively, and one “counter” for the number of electron-proton pairs on the Q_b_. With zero, one, or two electron proton pairs on the Q_b_ and the other four sites either empty or occupied, a single RC alone can already be in 48 different states. In a traditional state-based model all these 48 states have to be included, in principle, and updated simultaneously for a complete description of the state vector of a single RC. With more protein types and more complex models, the number of possible states will grow exponentially. For traditional approaches, rule based setup tools have been developed to help with this tedious and error-prone setup of, for example, receptor phosphorylation in signaling cascades [23], [24]. In our model, however, there is no need to explicitly enumerate or even to define these states—we only have to check the occupancies of a few nearby sites to determine whether a given reaction may occur during the next time step.

Even though our approach is not optimized for numerical efficiency, the fast dynamics of a complete vesicle during a flash experiment can be simulated close to realtime at a time resolution of Δ*t* = 1 microsecond on a current desktop computer. This is fast enough to perform even an extensive multi-parameter optimization on a small cluster of workstations. The largest optimizations performed in this study took about ten days on 40 CPUs. This, however, is still much less time than what it took to compile the data, set up the simulation model, or to analyze the results.

#### Modeling the Cytochrome *bc*_1_ Complex

**Figure 2:**
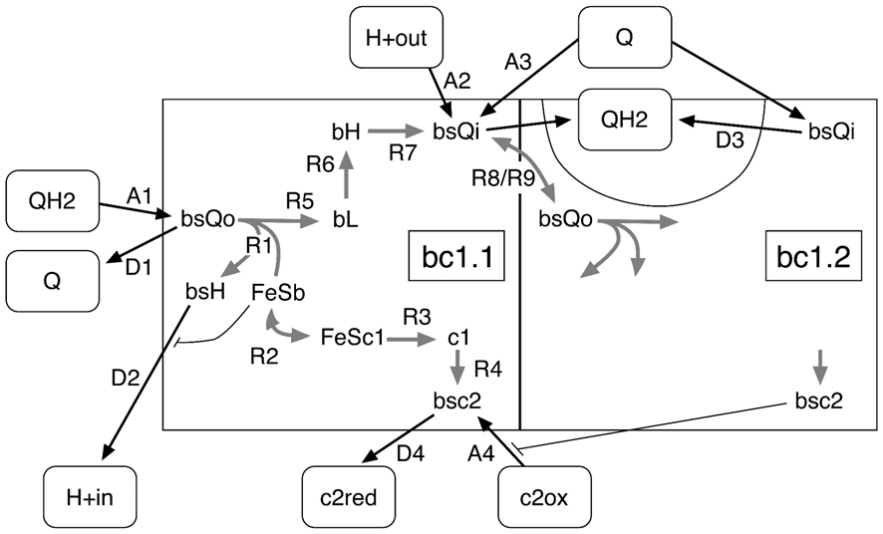
Reactions Used to Model the Dimeric Cytochrome *bc*_1_ Complex. Shown are only the reactions for the left half of the dimer (bc1.1). For the right half (bc1.2), only those reactions are given which are related to the left half of the dimer. In the simulation, the same set of reactions is considered for bc1.2 as for bc1.1. The rounded rectangles denote the metabolite pools. Association and dissociation reactions are indicated by black arrows, while the internal reactions are represented by dark grey arrows.

In our first model, most of the internal reactions of the cytochrome *bc*_1_ complex were lumped together into two reactions [4]. To be able to describe experimentally accessible data such as the oxidation state of the *c*_1_ domain, the current version now explicitly models the different pathways of the two electrons away from the QH2 docked into the Q_o_ site, see figure 2. For the first electron, we included the heme of the *c*_1_ domain and the hinge motion of the Rieske iron-sulphur domain (FeS) between the *b* and the *c*_1_ domains (positions FeSb and FeSc1 in figure 2, connected by the reactions R2). For the second electron, we added the hemes *b*_L_ and *b*_H_ on its way from Q_o_ (indicated by the binding site bsQo in figure 2) to Q_i_ (bsQi). Also, the proton release into the vesicle is now coupled to the gating motion of the FeS domain (reaction D2) [25]. The rates for this proton release [26] and for the electron transfer between *b*_H_ and *b*_L_, which are the major charge transfer steps against the transmembrane potential (reactions D2 and R6 in figure 2), are modeled to decrease exponentially with increasing transmembrane potential ΔΦ. We used the same scaling for both reactions. In the current model, the kinetics of the *bc*_1_ complex are thus modeled by four association and four dissociation reactions connected to external metabolite pools (A1 to A4 and D1 to D4, respectively, in figure 2), by seven internal electron and proton transfer reactions (R1 to R7), and by the two quinone (quinol) exchange reactions between the Q_i_ and the Q_o_ sites of the two dimer halves (R8 and R9). This set of reactions reproduces the Q-cycle proposed by Crofts [12].

Further details and the implementation of the other proteins, i.e., of the LHC, the RC, and the ATPase are given in [4]. These proteins are also not modelled with all known details. The design criterion was to incorporate all reactions that are required to reproduce the available experimental data. Thus, when new experiments performed under different conditions or on different timescales would be added to the current set, the protein models most probably would have to be updated, too.

#### The Transmembrane Potential ΔΦ

The second modification of our earlier model [4] addresses the calculation of the transmembrane potential across the vesicle membrane. Previously, only the Nernst relation had been used, which relates the transmembrane potential ΔΦ_chem_ to a proton concentration (or pH) difference across the membrane [27]:
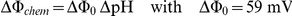
The standard value of the conversion factor ΔΦ_0_ was derived from a thermodynamic analysis at a planar wall separating two infinitely large half spaces [28]. However, such a configuration hardly applies to a finite-sized spherical vesicle. Moreover, the inner volume of a chromatophore vesicle is so small that one cannot assume a continuous, macroscopic density. In fact, a situation with pH = 7 in a typical chromatophore vesicle of 45 nm outer diameter corresponds to one twentieth of a free proton inside the vesicle. In this volume, a single free proton already creates a pH = 4.1 and four protons would be enough for a typical transmembrane potential of ΔΦ_chem_ = 200 mV. To make as few assumptions as possible about the driving force of a proton gradient in this small vesicle, we treated ΔΦ_0_ as a freely adjustable conversion factor that relates the concentration of free protons inside the vesicle to a contribution ΔΦ_chem_ to ΔΦ. ΔΦ_0_ was then tuned in the optimization such that the kinetic response of ΔΦ matched the experiments as well as possible.

In real biological vesicles, however, the lipids of the membrane and the solvent exposed protein surfaces contain a large number of titratable groups. These allow for *averaged* fractional proton numbers in the vesicle, eliminating the unrealistic discretization steps in the pH difference due to the very small number of free protons. To account for such titratable groups inside the vesicle, a special pseudo-protein was included in the simulations which has *N*_p_ proton binding sites with a given pK value. Each of these *N*_p_ proton binding sites is protonated with a probability
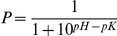
As a conservative estimate, we placed one titratable residue on each of the transmembrane proteins, which yields *N*_p_ = 80 for our model chromatophore. Assuming, for example, a pK = 4.5, which is close to the pK = 3.8 value of aspartic and glutamic acid residues which are often found on solvent exposed protein surfaces, 76 protons are then required to generate ΔΦ_chem_ = 200 mV. Only four of these 76 protons will be free in solution whereas the other 72 are bound to the titratable groups. In the optimization (see below), pK was varied to check for its effect. Tests showed that for *N*_p_>80 the dynamic behavior of the vesicle did not change anymore. Also, as will be shown below, for increasing *N*_p_ the importance of ΔΦ_chem_ actually decreases relative to the electric contribution ΔΦ_cap_ which accounts for the charges of the protons themselves.

This second contribution to ΔΦ arises not only from the charges of the protons inside the vesicle but also from those bound to the titratable residues and from the charges displaced perpendicular to the membrane in the proteins. Similar to ΔΦ_chem_ this contribution was calculated from the total number of charges *N*_q_ and a freely adjustable conversion factor Δ*U*_0_ for the transmembrane potential created by a single charge.
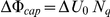
In this capacitor model Δ*U*_0_ describes the (yet to be determined) effective inverse capacitance of the complete vesicle without any assumptions about its form or dielectric properties. For comparison, Feniouk et al. used the specific capacitance per area of 1 µF cm^−2^ of a planar membrane to estimate the electrical capacity of a typical vesicle as 5×10^−2^ fF [29]. With this capacitance we would get Δ*U*_0_ = 3.2 mV/e and the 76 H^+^ estimated above for ΔΦ_chem_ = 200 mV lead to an additional ΔΦ_cap_≈240 mV.

Based on observed relaxation times, Feniouk et al. concluded that the chemical proton buffering capacitance is about 3.5 times larger, i.e., less important for ΔΦ than the electric contribution. A slightly smaller ratio between ΔΦ_cap_ and ΔΦ_chem_ was reported by Klamt et al., who found that two thirds of the protonmotive force are due to the charges and one third stems from the proton density gradient [17]. Both these observations are consistent with our findings that ΔΦ_cap_ is much more important for the chromatophore kinetics than ΔΦ_chem_ (see below). Here we did not attempt to fix the conversion factors ΔΦ_0_ and Δ*U*_0_ from a physical model but tried to find values for them that allowed reproducing the time dependent experiments in the best way. Only then can we interpret the obtained results in thermodynamic terms.

Due to their different physical origins, the two terms contributing to ΔΦ scale differently with an increasing number of protons in the vesicle. While the chemical contribution ΔΦ_chem_ increases logarithmically with the number of free protons, ΔΦ_cap_ grows linearly with the total number of charges. Consequently, for a few protons in the vesicle, ΔΦ_chem_ is the main contribution, while for larger numbers of protons or charges displaced in the activated proteins, ΔΦ_cap_ dominates and the accurate description of ΔΦ_chem_ becomes less important. For the bacterial ATPase, it does not matter whether the proton driving force is generated via ΔΦ_cap_ or via ΔΦ_chem_ [30]. In contrast, for electron or proton transfer processes perpendicular to the membrane in the proteins, only the electric field resulting from ΔΦ_cap_ matters. With the numbers estimated above, we can consequently expect that for metabolically relevant values of the total transmembrane potential ΔΦ = ΔΦ_chem_+ΔΦ_cap_≈200 mV, where the ATPase already runs at full speed, the dynamic response of the photosynthetic chain is mainly determined by ΔΦ_cap_.

### Parameter Optimization

The optimization of the rate constants was started from a set of rates compiled from experimentally determined reaction rates and estimates from the steady state reconstruction [13]. For those reactions where no direct information was available, we used estimates based on similar reactions in other types of proteins or sometimes even an “educated guess”. We found that this approach works very well for the purpose of initializing the search. This especially applies to combined reactions which describe a sequence of reaction steps taking place in the real protein. One example is the exciton induced electron transfer through the RC, which consists of three consecutive steps with each step being about one order of magnitude slower than the previous one [31].

#### Experiments and Parameters selected for the Optimization

To determine the unknown kinetic parameters, we ran molecular stochastic simulations according to seven time dependent measurements on the photosynthetic apparatus of *Rb. sphaeroides* reported by Barz *et al.* [5], [6]. Those experiments investigated the role of the protein PufX and covered a wide range of time-scales from fast single-flash experiments over multi-flash scenarios to quasi steady state conditions. Because PufX lacking mutants of *Rb. sphaeroides* are not photosynthetically competent, the bacteria were grown semiaerobically and then incubated anaerobically in the dark before the actual experiments started. Some experiments were performed on dark adapted vesicles which had been extracted from cells disrupted in a French press. For all simulations, both of the whole cell and of the vesicle scenarios, we used the same standard vesicle of 45 nm outer diameter with ten dimeric core complexes composed of one LHC and two RCs, ten *bc*_1_ dimers, and one ATPase. The model vesicle also contained as carriers 200 quinones and 20 cytochrome *c*_2_ as well as 80 titratable residues. All scenarios were started from a dark adapted initial state. Consequently, the cytochrome *c*_2_ pool was initially completely reduced and only 15 of the 200 quinones were oxidized. To get reproducible statistics, the single- and multi-flash simulations were repeated 40 times for every parameter set and their outputs were averaged before running the analysis.

We now shortly describe the selected experiments and point out how special features of the scenarios were implemented in the simulations. The letters A or B are used to denote whether the experiment was reported in [5] or [6], together with the respective figure number and an additional label indicating the measured quantity. For the parameter optimization we used the data from the PufX containing mutant which closely resembles the wildtype. The chromatophores are so small that the diffusion of the cytochrome *c* throughout the vesicle interior and of the quinones across the complete membrane area are much faster than the respective binding and unbinding rates [13]. Therefore, any spatial arrangement of the proteins can be safely ignored here.

**A7_ΔΦ** and **A7_cytc**: Figure 7 of [5] reports the spectroscopically determined electric component of the transmembrane voltage and the cytochrome *c* oxidation state after one single-turnover flash in dark-adapted anaerobic bacteria. The purpose of this experiment was to show that PufX is not an integral part of the electron transport chain as even without PufX cyclic electron transport can occur. Consequently, the experimental results were basically the same for wild-type and mutant strains. The experiment was performed on anaerobically incubated dark adapted cells, which we modelled by a single dark adapted vesicle. In the simulation, the cytochrome *c* oxidation was determined as the total number of oxidized *c*_2_ and of oxidized *c*_1_ domains of the *bc*_1_ and rescaled for comparison with the arbitrary units of the experimental data. The electric part of the transmembrane potential was measured via absorption difference spectra of the carotenoids of the LH2 [7]. The strength of the signal depends on the number of the LH2, which may be different in the various strains. The experimental data were therefore normalized to the fast first step induced by the charge transfer in the RCs and thus provides absolute values for the timing and relative values for ΔΦ_cap_ and the cytochrome *c* oxidation state. In the simulation also the chemical part of ΔΦ was included. The flash was simulated by a light intensity of 1500 W/m^2^ for 0.1 milliseconds, which is fast and strong enough to initiate a single electron transfer from the special pair to Q_b_ in nearly all RCs. Note that due to the stochastic nature of the simulations there is always the chance that one of the RCs does not trigger even upon an extremely strong flash. At the chosen intensity of the single flash, most RCs end up in the semiquinone state with a single electron-proton pair on the initially oxidized Q_b_. The total simulation time was 0.3 seconds using a timestep of 2 microseconds where the flash occurred after 30 milliseconds. The 40 independent runs per parameter set took about 50 seconds to simulate and analyze as described below.

**A8_ΔΦ** and **A9_cytc**: While PufX does not affect single turnover electron transport, it is required for continuous turnover of the photosynthetic apparatus as demonstrated by the next experiment. The two quasi steady state measurements shown in figures 8 and 9 of reference [5] report ΔΦ_cap_ and the cytochrome *c* oxidation state of intact anaerobic cells during a nine seconds long continuous illumination. Without PufX, ΔΦ_cap_ and the cytochrome *c* oxidation level shortly rise upon illumination and then decay again as in the single flash experiment A7, whereas in the wildtype both signals saturate after about two seconds while the light is on. Again, ΔΦ_cap_ was rescaled to the first fast step and the cytochrome *c* oxidation given in arbitrary units. Here, a single closed vesicle was simulated for 12 seconds using a timestep of 20 microseconds. After one second in the dark the illumination was switched on for nine seconds with an intensity of 90 W/m^2^. This intensity is higher than the reported value of 50 W/m^2^ in the experiment to ensure that the light is still saturating when the absorption cross section of the LHCs is reduced during the optimization. The five simulation runs for every parameter set took about 40 seconds including analysis.

**B1_Q**: To show that the RC is fully functional in a PufX deficient mutant, Barz et al. probed isolated chromatophores with a series of four single-turnover flashes spaced 1 s apart [6]. After each flash one electron-proton pair is transferred onto the Q_b_ quinone bound to the RC. When loaded with a second electron-proton pair after the next flash, the quinol detaches from the RC and is replaced by another quinone. As this exchange is fast compared to the spacing between the flashes, semiquinone oscillations can be observed spectroscopically. In this experiment the *bc*_1_ complexes were inhibited by antimycin A, which blocks electron transfer from heme b_H_ onto the quinone bound at the Q_i_ site. Additionally, N,N,N′,N′-tetramethyl-p-phenylenediamine was added in the experiment, which efficiently reduces the RC special pair and oxidizes Q_b_^−^ with a slow time constant of minutes that is negligible here. This scenario, in which only the state of the RCs was probed, was simulated with a vesicle which only contains the LHCs and the RCs. The pool of reduced cytochrome *c*_2_ was set to a constant concentration to mimick the fast re-reduction of the special pair after each of the flashes. In the experiment the chromatophore density was adapted such that each flash induced a single turnover in about 90% of the RCs. For a similar turnover probability in the simulation the flash was set to 4000 W/m^2^ for 0.2 ms.

**B6_P** and **B6_cytc**: These two scenarios are again different measurements performed during the same experiment, where the oxidation states of both the special pair bacteriochlorophylls of the RCs (P) and of the total cytochrome *c* content in anaerobic dark-adapted cells were monitored during a train of 16 flashes spaced 20 ms apart (figure 6 of reference [6]). After the first flashes, a dynamic equilibrium is established between the pulsed excitation of the RCs and the turnover of the subsequent steps in the photosynthetic chain. Consequently, the flash intensity has to be so low that the single ATPase can utilize all the protons pumped into the vesicle. From the maximum turnover of the ATPase of 400 H^+^/s we find that no more than 100 QH_2_ per second may be produced by the RCs under an excitation every 20 ms, i.e., with a frequency of 50 s^−1^. Thus, each flash may not excite more than four of the 20 RCs. To obtain such a 20% excitation probability in the simulation we used a comparably low light intensity of 400 W/m^2^ for 0.1 milliseconds. The complete dark-adapted vesicle was simulated for 0.4 seconds with the first flash occuring after 50 milliseconds. The cytochrome *c* oxidation state was again determined from the *c*_2_ and the *c*_1_ of the *bc*_1_. The 40 iterations plus the analysis took about 80 seconds on a PC using a simulation timestep of 2 microseconds.

**BC1**: In addition to these experiments, we tested for each parameter set the steady state throughput of the cytochrome *bc*_1_ complex against the experimentally observed maximal enzymatic turnover at vanishing ΔΦ [32]. For this a single *bc*_1_ dimer was simulated for 20 seconds using a timestep of 20 microseconds with the pools of Q, QH_2_, and oxidized cytochrome *c*_2_ set to fixed concentrations. After the simulation the number of reduced cytochrome *c*_2_ was used to determine the turnover. This test, which was labelled BC1, took about half of a second to perform.

The total time required to run all the scenarios A7, A8+9, B1, B6, and BC1 for one parameter set was about three minutes.

Together with the numbers of the proteins, the geometric properties of the vesicle, and the initial conditions, i.e., the initial pool occupation numbers, the model contains 44 rate constants and parameters relevant for the kinetics. Out of these 44, we selected 25 parameters for the optimization. These includes all parameters of the LHC and the RC, respectively, 13 of the 19 parameters of the *bc*_1_ complex, two of the nine constants related to ΔΦ, and one of the two parameters for the titratable residues. These parameters are listed together with their optimized values in table 1. Not included in the optimization were the fast internal electron transfer reactions in the *bc*_1_, the weights of the different types of charges contributing to ΔΦ_cap_, and the number of titratable residues. Independent tests showed that these parameters have only a very weak or no influence at all on the dynamic behavior of the complete vesicle. Their (fixed) values are listed in table S1. The ATPase was implemented as described in reference [4] and did not have any adjustable parameters.

**Table 1:** Optimized parameter values <*P*> with their ranges *P*_min_…*P*_max_.

parameter	units	<*P*>	*P*_min_…*P*_max_	*P*_min_/*P*_max_
LHC::σ	m^2^ W^−1^ s^−1^	6.22	6.02…6.42	0.94
LHC::*N*_0_	1	1.31	0.81 … 2.13	0.38
LHC::*k*_D_(E)	s^−1^	1.9 * 10^3^	(1.1…3.8) * 10^3^	0.29
RC::*k*_on_(E)	s^−1^	2.4 * 10^6^	(1.2…4.5) * 10^6^	0.27
RC::*k*_on_(H^+^)	nm^3^ s^−1^	1.4 * 10^8^	(1.3…1.6) * 10^8^	0.81
RC::*k*_on_(Q)	nm^2^ s^−1^	6.0 * 10^4^	(4.4…8.1) * 10^4^	0.54
RC::*k*_off_(QH2)	s^−1^	87	70…108	0.65
RC::*k*_on_(c2red)	nm^3^ s^−1^	9.2 * 10^5^	(7.3…11.5) * 10^5^	0.63
RC::*k*_off_(c2ox)	s^−1^	2.2 * 10^3^	(1.6…3.0) * 10^3^	0.53
bc1::kon(QH2@Qo)	nm^2^ s^−1^	1.2 * 10^4^	(0.79…1.7) * 10^4^	0.46
bc1::koff(Q@Qo)	s^−1^	28.3	26.3…30.4	0.86
*bc*_1_::*k*_tr_(Q:Q_o_ = >Q_i_)	s^−1^	4.9 * 10^3^	(3.6…6.7) * 10^3^	0.54
bc1::kon(Q@Qi)	mm^2^ s^−1^	6.7 * 10^5^	(4.5…10) * 10^5^	0.45
bc1::koff(QH2@Qi)	s^−1^	86	68…110	0.62
*bc*_1_::*k*_tr_(QH2:Q_i_ = >Q_o_)	s^−1^	3.8 * 10^3^	(2.6…5.5) * 10^3^	0.47
*bc*_1_::*k*_on_(c2ox)	nm^3^ s^−1^	9.4 * 10^6^	(6.3…14) * 10^6^	0.47
*bc*_1_::*k*_off_(c2red)	s^−1^	6.0 * 10^3^	(3.3…11) * 10^3^	0.30
bc1::koff(H+@Qo)	s^−1^	2.4 * 10^4^	(1.3…4.3) * 10^4^	0.30
*bc*_1_::*k*_tr_(FeS:b = >c)	s^−1^	3.9 * 10^3^	(3.1…5.1) * 10^3^	0.61
*bc*_1_::*k*_tr_(FeS:c = >b)	s^−1^	2.8 * 10^3^	(2.2…3.6) * 10^3^	0.61
*bc*_1_::*k*_tr_(e:b_H_ = >Q_i_)	s^−1^	7.7 * 10^3^	(5.0…12) * 10^3^	0.42
*bc*_1_::Φ_0_	mV	102	83…114	0.73
ΔΦ::U_0_	mV/e	10.3	9.5…11	0.85
ΔΦ::ΔΦ_0_	mV/pH	10	7.6…13.7	0.55
PR::pK	1	4.84	3.9…5.9	0.66

The sensitivities of the complete set of simulations with respect to each of the parameters are expressed via *P*_min_/*P*_max_. These values were determined from the 1000 best parameter sets as explained in the text.

#### Scoring the Parameter Sets

The quality of a given parameter set was judged from the overall agreement between the simulation results and the experiments. For this, an individual score *s*_i_ was determined for each of the eight scenarios from the normalized squared distance between the simulation results *x*(*t*_i_) at equidistantly spaced time points *t*_i_ and a fit function to the experimental data at the same time points *f*(*t*_i_):
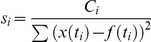
For convenience, the normalization constants *C*_i_ were adjusted such that the scores for the best parameter sets were on the order of one for each of the experiments. However, their actual values are not important, as the same *C*_i_ were used for all parameter sets. They only affect the overall scaling of the scores without changing their relative ordering. For scoring the individual simulations, we used a fit to the experiment rather than the raw experimental data points. This was done for two reasons. First, the experimental data is noisy, i.e., each of the discrete data points has an unknown deviation from the correct value. Continuous fit functions composed from constant, linear, and exponential terms allowed us to average a smooth curve through the noisy data. Second, the data points are often measured at rather large or non-equal time intervals. With the continuous fits the simulation could be scored at the most convenient time intervals. The scoring functions for the chosen experiments are listed in supporting Text S1.

The overall master-score *S* of a parameter set was then determined as the product of the individual scores *s*_i_. Multiplying the individual scores ensures that a parameter set has to perform well in all simulations in order to achieve a good overall score in the optimization.

The optimal value for each individual parameter was then determined by computing the logarithmic average <*P*> and variance σ of a parameter *P* from the 1000 highest-scored parameter sets among the 32800 different parameter sets:
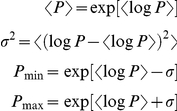
With this logarithmic averaging, the sensitivity of the model with respect to the value of a given parameter can be expressed as the respective ratio of P_min_/P_max_.

### Estimating the Number of *bc*_1_ Complexes

In the previous reconstruction of a typical chromatophore vesicle [13] the steady state throughputs indicated that already three cytochrome *bc*_1_ dimers could suffice to supply the ATPase with enough protons to run at full speed. From wet lab experiments, however, a ratio of about one *bc*_1_ dimer per dimeric core complex was found, i.e., about ten *bc*_1_ complexes per vesicle [5]. As the *bc*_1_ can only use the limited amount of energy stored in the electron proton pairs on the QH2 to pump the protons against the transmembrane potential ΔΦ, its throughput decreases with increasing ΔΦ. Thus, in a steady state scenario it does not make a difference whether the model includes only three or ten or even more *bc*_1_ complexes as their turnover will be reduced once a certain transmembrane potential is reached. In a dynamic scenario, however, the response of ΔΦ to a rapid increase of the illumination will be faster in a vesicle carrying more *bc*_1_ complexes.

Therefore, we first investigated how the number of *bc*_1_ dimers affects the non-equilibrium dynamic response of a chromatophore vesicle during the single flash experiment A7 introduced above [5]. This experiment monitored spectroscopically the electric contribution ΔΦ_cap_ to the transmembrane potential and the oxidation state of all cytochrome *c* after a short flash in anaerobic cells of *Rb. sphaeroides* PUFC/g mutants, which resemble the wild type (figures 7A and 7B, respectively, of [5]). In the dark adapted cells, ΔΦ_cap_ was initially zero and increased very fast with the flash with a biphasic rise and a subsequent exponential decay. The first very fast rise of ΔΦ_cap_ is due to the electrons displaced in the RCs and the second slower rise is due to the protons pumped into the vesicle once the first QH2 arrive at the *bc*_1_s. Simultaneously, the cytochrome *c* oxidation state showed a short peak that decayed on the millisecond timescale of the second rise of ΔΦ_cap_.

We mimicked this experiment with varying numbers of *bc*_1_ dimers of *N*_bc1_ = 3 … 14 in the simulations to optimize four parameters and observe how their respective optimal values changed with *N*_bc1_. These four parameters are *bc*_1_::Φ_0_, which describes the decrease of the *bc*_1_ pumping activity with increasing total ΔΦ, the transmembrane voltage per ΔpH unit, ΔΦ::ΔΦ_0_, the transmembrane voltage per unit charge in the vesicle, ΔΦ::U_0_, and the pK value of the 80 titratable residues that are placed in the vesicle, PR::pK. PR::pK and ΔΦ::U_0_ determine the buffering capacity of the vesicle, whereas the number of *bc*_1_s and their cut-off voltage *bc*_1_::ΔΦ_0_ determine the proton pumping rate of the *bc*_1_s. The parameters ΔΦ::ΔΦ_0_ and ΔΦ::U_0_ finally control the chemical and the electric contributions to the transmembrane potential which are denoted by ΔΦ_chem_ and ΔΦ_cap_, respectively. Together with *N*_bc1_, these four parameters thus determine how fast ΔΦ_cap_ and the cytochrome *c* oxidation state may change. All other kinetic parameters of the proteins were based on the first version of the stochastic model as reported in [4]. Later tests with the new optimized kinetic parameters gave essentially the same results. This means that at least for the parameters that are important for this specific experiment our initial estimates were good enough to identify a realistic *N*_bc1_.

**Figure 3:**
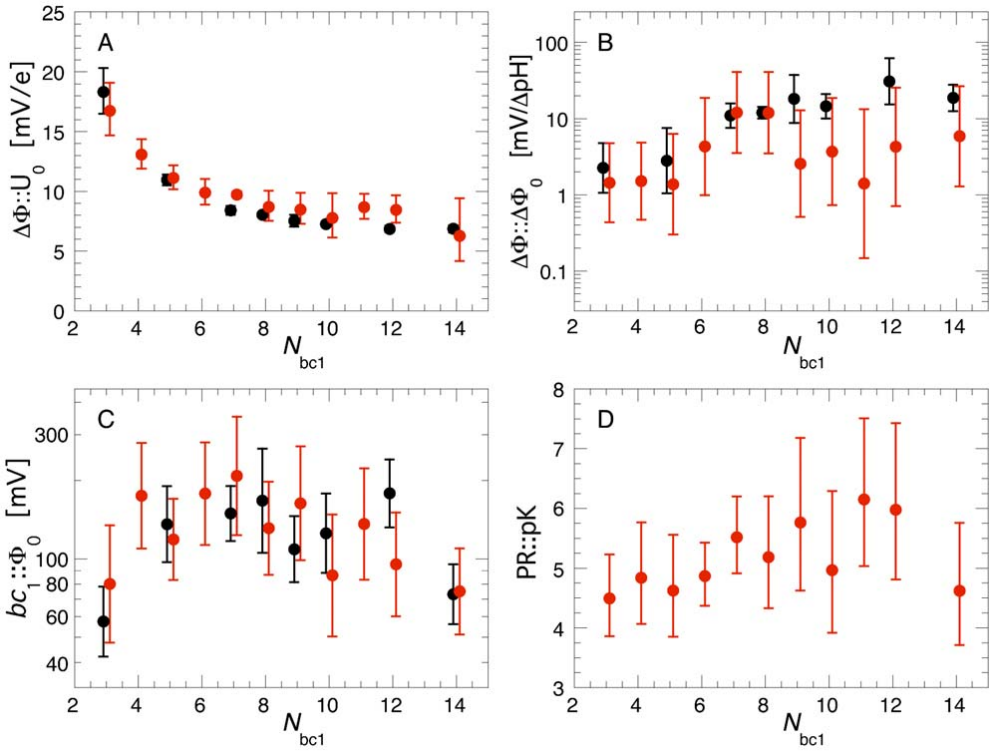
Optimal Parameter Values vs. Number of *bc*_1_ Dimers. Average parameter values from the best 100 individuals (out of the 840 individual optimizations) vs. the number of *bc*_1_ complexes *N*_bc1_. In the two optimizations shown, we simulated the course of the transmembrane voltage ΔΦ_cap_ and of the cytochrome *c* oxidation state during a single flash experiment with dark-adapted bacteria (see text for further explanations). The black data points show the results from the first optimization round where ΔΦ::U_0_, ΔΦ::ΔΦ_0_, and *bc*_1_::Φ_0_ were optimized. In the second round (red symbols) also PR::pK was considered.

With this simulation setup, two sets of evolutionary parameter optimizations were performed where *N*_bc1_ was varied from 3 to 14. In the first set, ΔΦ::U_0_, ΔΦ::ΔΦ_0_, and *bc*_1_::Φ_0_ were optimized. In the second set, PR::pK was included as a fourth parameter. In each individual optimization run, 21 iterations were performed with 40 individuals per generation, i.e., 840 parameter sets were tested for every *N*_bc1_. In all optimizations, the scores converged after about five iterations (see figure S1). For the analysis the parameter sets were sorted according to their master score. Average values of the master scores and of the four parameters were determined from the 100 best of the 840 parameter sets. Both optimization runs gave comparable scores and parameter values. The resulting averaged scores increased from *N*_bc1_≥4 to *N*_bc1_ = 8 and slightly decreased again for *N*_bc1_≥9 (see figure S2). This means that based on the master scores alone *N*_bc1_ = 8 would be the optimal number of *bc*_1_ complexes for the modeled vesicle. Figure 3 shows how the optimal values for the four parameters ΔΦ::U_0_, ΔΦ::ΔΦ_0_, *bc*_1_::Φ_0_, and PR::pK vary with *N*_bc1_. For *N*_bc1_ = 3, ΔΦ::U_0_ is rather high and converges to lower values for larger *N*_bc1_. The decrease with *N*_bc1_ means that the larger proton pumping rate resulting from more *bc*_1_ complexes has to be compensated by an increased electrical buffering capacity, i.e., a smaller ratio of voltage change per unit charge. The convergence for *N*_bc1_≥8 indicates that a larger number of *bc*_1_ dimers represents a more favourable “stable” model in terms of kinetic constants.

The large standard deviations for ΔΦ::ΔΦ_0_ indicate that the actual value of this parameter (which represents the conversion factor from the pH gradient across the membrane into an equivalent electrical potential with the same driving force) is rather irrelevant for a good agreement between the experiment and the respective simulation in this fast transient scenario. Instead of the usually used 59 mV/ΔpH, the values ranged between 1 and 20 mV/ΔpH here. With these values, ΔΦ_chem_ contributes less than 30% to the total ΔΦ. In fact, simulations that completely neglected ΔΦ_chem_, i.e. ΔΦ::ΔΦ_0_ = 0, could reproduce the experimental data with comparable accuracy. As ΔΦ::ΔΦ_0_ increases with *N*_bc1_ from too small towards more realistic values, one would choose *N*_bc1_ as large as possible based on the behavior of this parameter, too.

The third parameter, *bc*_1_::Φ_0_, regulates at which value of ΔΦ the pumping rate of the *bc*_1_s is reduced. Its optimal value decreased with increasing *N*_bc1_. However, with the obtained values of *bc*_1_::Φ_0_≥80 mV, the throughput of the *bc*_1_s is only limited at larger ΔΦ while still allowing for a fast proton pumping at low ΔΦ. To prevent ΔΦ from rising too high during steady state situations, a smaller *bc*_1_::Φ_0_ should be preferable, again suggesting to use a larger *N*_bc1_. The pK value of the titratable groups, finally, depends only weakly on *N*_bc1_. Its trend to increase with *N*_bc1_ can again be understood as increasing the effective buffering capacity of the vesicle by an earlier onset of proton binding.

Based on these results, we chose to use ten cytochrome *bc*_1_ complexes for the typical vesicle in the further optimizations of the kinetic parameters of the photosynthetic apparatus. This value obtained from the dynamic simulations fits well with the stoichiometries of approximately one *bc*_1_ dimer per two RCs determined in wet-lab experiments [5] and is about three times larger than the minimal number estimated from the steady state throughputs [13].

### Optimized Parameter Values

After having fixed the stoichiometries of all proteins, we started the actual optimization of the kinetic parameters for the elementary reactions in the model vesicle. From the 44 parameters of the model, we selected 25 (see table 1). Not included in the optimization were, e.g., the numbers of protein copies which were set to fixed values, the vesicle size, and the details of the fastest reactions inside the *bc*_1_ complexes (see table S1). The parameters were optimized in two main runs which considered subsets of 15 and 12 of the 25 parameters, respectively. The two parameters ΔΦ::U_0_ and *bc*_1_::Φ_0_ were included in both optimization runs, in which they converged to about the same values, differing by about 10%. These two main runs consisted of 41 generations of 800 individual parameter sets each. They were accompanied by several smaller optimization runs where, e.g., only the parameters of a single protein were optimized. In the smaller optimization runs the population sizes were reduced to 100…150, but the number of generations was kept the same. To within the statistical uncertainties, these independent optimizations of the parameters of the individual proteins confirmed the results from the two large optimization runs. In all optimization runs, reasonable parameter values were found within the first five to ten generations and further optimized in the subsequent iterations (see figure S3).

**Figure 4:**
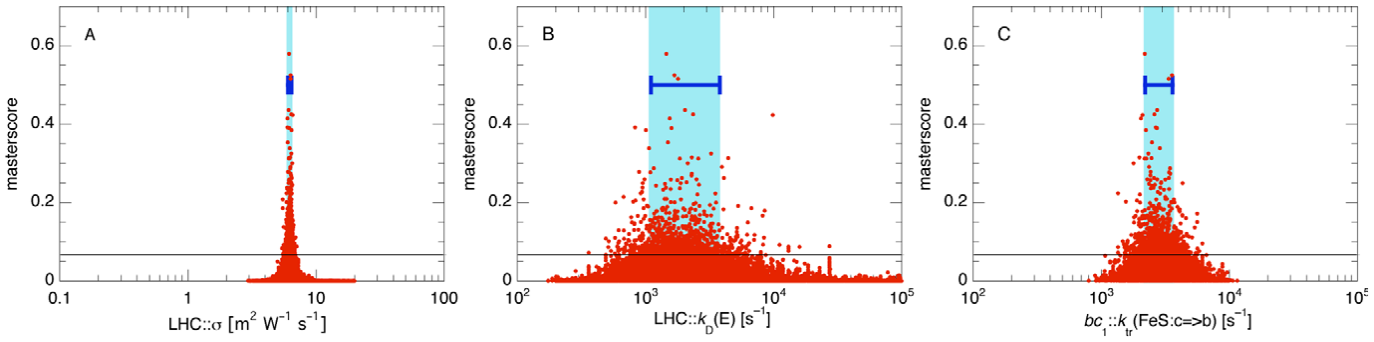
Extracting Optimal Parameter Values and Sensitivities. Projection of the master score on the values of three parameters: LHC::σ, the absorption cross section of the LHC (panel A), LHC::*k*_D_(E), the decay rate of excitons in the LHC (B), and *bc*_1_::*k*_tr_(FeS:*c* = >*b*), the kinetic rate for the hinge motion of the FeS domain of the *bc*_1_ complex from the *c*_1_ to the *b* domain (C). For these plots the master scores were rescaled such that the best score had a value of 1. For clarity, this best data point is not shown. In this figure, the best 1000 parameter sets had master scores ≥0.068, which is indicated by the thin horizontal line. The respective intervals for the optimized parameters obtained from all parameter values with scores above this threshold are denoted by the blue regions and the horizontal error bars.

To determine the optimal kinetic parameters for the individual reactions, the obtained multi-dimensional distributions of the master-scores were projected onto each of the individual parameters as shown in figure 4 for three representative parameters. According to panel A of figure 4 the good scores above 0.068 of the best 1000 parameter sets can only be achieved when the absorption cross section of the LHCs, LHC::σ, has a value very close to 6.3 m^2^ W^−1^ s^−1^. Even when this parameter was chosen correctly, low scores were still found when one or more of the other parameters were off their optimal values. On the other hand, panel B of figure 4 shows the projection onto the decay rate of unused excitons in the LHCs, LHC::*k*_D_(E), which could be varied over more than two orders of magnitude while still reaching scores above 0.068. Consequently, the actual value of this parameter plays a minor role for a good agreement between simulation and experiment. Panel C of figure 4 finally shows the distribution of *bc*_1_::*k*_tr_(FeS:*c* = >*b*), a parameter of intermediate sensitivity. Here, scores above 0.068 were obtained with parameter values varying within about one order of magnitude. In each case, the peak of the distribution of the master score gives the optimal value of this parameter, and the width of the distribution encodes the importance (sensitivity) of this parameter for a correct *in silico* description of the experiments.

The obtained optimal parameter values <*P*> and their sensitivities P_min_/P_max_ are listed in table 1. When good, high-scoring parameter values were found in a narrow range only, then *P*_min_ and *P*_max_ are very close to each other and the ratio *P*_min_/*P*_max_ is close to one (cf. figure 4A). Most parameters have a sensitivity *P*_min_/*P*_max_ of around 0.5, whereas this ratio can even be below 0.1 for the less sensitive parameters (as in figure 4B). For the two parameters ΔΦ::U_0_ and *bc*_1_::Φ_0_ which were included in both optimization runs, table 1 lists the respective averages obtained from the two runs. Generally, all parameter values that were previously known from experiment were found within their expected ranges which indicates that the optimization procedure could recover the correct parameter values.

### Reproducing the Experiments

With the optimized kinetic parameters (see table 1), the remaining kinetic parameters that were not included in the optimization process, and the stoichiometries of the proteins and metabolites (see table S1), our molecular stochastic model of the bacterial photosynthetic apparatus is now parametrized completely. In this section, we analyze how well the model with the optimized parameter set reproduces the individual experiments which span a kinetic range from the few milliseconds long signal of the cytochrome *c* oxidation state in response to a single flash in A7_cytc up to the three orders of magnitude slower quasi steady state scenarios of A8_ΔΦ and A9_cytc.

**Figure 5:**
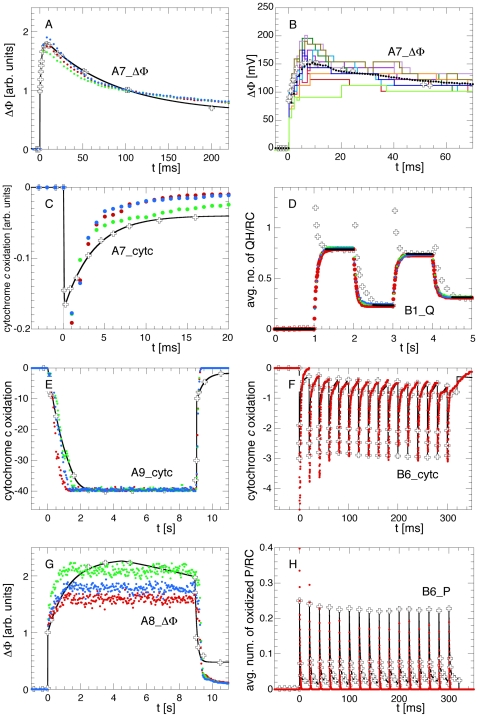
Experiments vs. Simulations with the Best Parameter Sets. Comparison between the experimental data [5], [6] (open crosses) and the simulations with the three best scored parameter sets (red, green, and blue points). Also shown are the fit functions used for scoring the respective observable (black lines, for details see supporting Text S1). For clarity, only a single simulation run is shown for B6_cytc and B6_P (panels F and H). Here, the other two traces are indistinguishable from the ones shown. The variation between these three traces reflects the variability of the results with the optimized parameters when the same scenario is simulated repeatedly. Panel B shows the statistical variations between individual runs with the optimized parameter set. It shows ten individual traces and the average of 40 simulations as used during the optimization.

Figure 5 compares the simulation results obtained with the three best parameter sets, which were all very close to the optimized values of table 1, to the respective experiments by Barz et al [5], [6]. Due to the stochastic simulation algorithm even the traces obtained from averaging over 40 individual simulations vary between repeated runs. The variations between the three runs shown in figure 5 are typical for the spread that is observed when the optimized parameters are used repeatedly. The overall agreement is quite remarkable. We will now discuss the individual panels and the remaining deviations between the simulation results and the experiments. These deviations mainly result from simplifications of the model and the simulation setup.

In the single-flash experiment **A7**, the biphasic rise and subsequent decay of ΔΦ_cap_ is well reproduced (**A7_ΔΦ**, panel A of figure 5). In this scenario, the decay of ΔΦ_cap_ is very sensitive to U_0_, i.e., to the electric capacitance of the vesicle. The corresponding cytochrome *c* oxidation state, **A7_cytc**, on the other hand, decays slightly too fast (panel C of figure 5). At first, this suggests that the related rate constants are too fast. However, the simulation only considered the photosynthetic apparatus located in the vesicles, whereas the experiment was performed on complete bacteria where the photosynthetic apparatus is also found in the inner membrane together with the proteins of the respiratory chain. Consequently, this fraction of the photosynthetic apparatus works against the larger combined cytochrome *c*_2_ pool of the photosynthetic and respiratory pathways, leading to a slower decrease of the cytochrome *c* oxidation. Secondly, in the simulation the light was switched on and off instantaneously while in the experiment the flashbulb takes some finite time to reach its maximal brightness before it cools down continuously. Without knowing the characteristics of the original experimental setup of Barz et al. [5], [6], we can only speculate about the actual illumination profile. A decay time of the flash on the millisecond timescale would suffice as well to explain the slower decay in the experiment.

For scenario A7_cytc panel B illustrates the stochastic nature of the simulations. It compares the average of 40 typical individual simulations with the optimized parameter values to the experiment and shows 10 of the 40 individual simulations results. In comparison to panel A, here the actual values of ΔΦ_cap_ are given, i.e., here the experimental data are scaled to the simulation instead of rescaling the simulations to the experiments as during the optimization process. The experiments were originially published in arbitrary units. Generally, the first step of ΔΦ_cap_, which originates from the electrons translocated in the RCs, was well reproduced in all individual runs while the subsequent rise due to the proton pumping of the *bc*_1_ exhibited strong variations from run to run. In some runs the *bc*_1_ pumped so slow that the protons could leave the vesicle via the ATPase before the additional contribution to ΔΦ could develop. However, in other runs all *bc*_1_s immediately pumped their two protons and the second rise of ΔΦ was as high as the first step. A similar variability can be found in the other scenarios, too.

In B1_Q (panel D of figure 5), the major differences between measurement and simulation are the spikes directly after the flash seen in the spectroscopic experiment. These are missing in the simulation because here the flash does not interfere with the determination of the number of bound QH. Consequently, we only used the flat parts of the experimental signal for scoring the simulation and neglected the intervals during the spikes. This experiment is most sensitive to the quinone association and dissociation rates at the RCs and, of course, to the light absorption cross section LHC::σ of the LHCs.

In the quasi steady state scenarios of **A8_ΔΦ** and **A9_cytc** (panels E and G, respectively, of figure 5), the dynamics of the cytochrome *c* oxidation were reproduced well. The only noticeable difference is that at the onset of the nine seconds long continuous illumination the oxidation of the initially fully reduced cytochrome *c* takes place about twice as fast in the simulation than in the experiment. The speed of this transition could have been slowed down in the simulation by decreasing the light intensity, but we chose to stick to the description of the experiment which states that the light intensity was “saturating”, i.e., that the RCs work at their maximal speed [6]. With less light this could not have been ascertained in the simulation for all values of the varied parameters. Independent of the absolute value of the light intensity also the re-oxidation of the cytochrome *c*_2_ at the end of the illumination was too fast. The main reason for these differences is that we simulated a single vesicle whereas the experiment was performed on complete cells in which a large fraction of their photosynthetic apparatus is located in the inner membrane together with the respiratory chain. Especially the final slow decay of the cytochrome *c* oxidation after *t* = 9 s in the experiment points to a spatially extended pool of cytochrome *c*_2_ so that diffusion must be considered explicitly (in contrast to the vesicle interior where diffusion is so fast that it can be neglected). For the complementing observation **A8_ΔΦ**, the differences between simulation and experiment were more pronounced. In the simulation, ΔΦ_cap_ increased with two phases as in A7_ΔΦ and then saturated within about one second as expected. In the experiment, ΔΦ increased slower and then even began to decay again while the light was still on. Barz et al. noted that they could not explain this slow transient overshooting and they reported that ΔΦ finally reached a steady state level after some 20 seconds of continuous illumination [6]. Consequently, there must be some slow charge relaxation processes present in the experiment which were not identified and thus could not be included in our simulation. On the other hand, the decay of ΔΦ after the illumination took place with about the same time constant both in the simulation and in the experiment.

For the multi-flash experiments **B6_cytc** and **B6_P** (panels F and H, respectively, of figure 5), the main difference between the experiments and the simulation is that the height of the flash-induced spikes decreased during the first few cycles while their amplitude was roughly constant in the experiment. This indicates that either the flashes were too strong or that the turnovers of either the RCs or of the *bc*_1_ were slightly too slow in the simulations to sustain the re-reduction of the RCs. Here again, some of the parameters related to the turnover of the RCs and the *bc*_1_ complexes are a compromise that is too slow for the fast flash experiments but too fast for the quasi steady state situations of A8_ΔΦ or A9_cytc. Whereas the simulation reproduced the time constant for the decay of the cytochrome *c*_2_ oxidation, the special pairs were reduced much too fast. This may be explained from the actual implementation of the RC in the simulation model. There, a reduced cytochrome *c*_2_ that is docked to an RC with an oxidized special pair delivers its electron to the special pair in the next time step after the *c*_2_ has bound, i.e., within less than one microsecond, whereas in reality this transfer may take longer. In the future, a separate rate constant for this electron transfer may lead to more precise results.

Overall, one can state that the experimental observations, which cover a range of timescales spanning three orders of magnitude, were reproduced well with the current model of the photosynthetic apparatus with the optimized parameters. The main difficulty, however, was that the experiments were partly performed on isolated chromatophore vesicles and partly on complete cells whereas our model consisted of a single chromatophore only.

### Biological Findings

From the systemically optimized parameter values we could derive several interesting biological findings for the molecular level of the model. Some of these findings confirm what had been known previously whereas other conclusions could not have been drawn from the experimental evidence alone.

**LHC**: we found that the decay of the excitons has to be included in the description. The best agreement with the experiments was achieved with a lifetime of about 0.5 ms. However, the actual parameter value was not so important. Such a lifetime is unrealistically long for the very fast kinetics of these electronic excitations which take place on pico- and nanosecond timescales. This shows one limitation of our model. In all other proteins of the photosynthetic pathway real particles are processed. Therefore a simulation timestep on the order of microseconds is fast enough to resolve these processes and is still numerically efficient enough. Because the exciton dynamics are neither rate limiting nor could be resolved in the current set of experiments, it was sufficient to use only three effective reactions to describe the processes in the LHCs. These are the photon capture, the bleaching at high light intensities, and a decay reaction which prevents that unused excitons “pile up” in the LHCs. To reproduce the fast flash experiments this effective reaction has to be much faster than the combined turnover of the two RCs of the dimeric core complex and, to achieve the high observed quantum efficiency of ≥90% of the LHC-RC units, much slower than the exciton transfer to the RCs. The exciton transfer, however, cannot take place faster than one simulation timestep which is on the order of a microsecond. This shows that when experiments that can resolve the very fast internal dynamics of the LHC will be added to our set of experiments the rates will become more realistic as a much shorter timestep will then be required and most probably more detail has to be included in the LHC model.

**RC**: For the RCs we determined the steady state throughputs with the optimized parameters. One RC then handles 12.5 photons per second, i.e., oxidizes 12.5 *c*_2_ per second. Interestingly, after the optimization the rate limiting reaction was the proton uptake from the cytoplasm. The rate limiting parameter was identified by observing how the steady state throughput changed when each parameter is scanned individually. At pH = 6.8 the obtained RC::*k*_on_(H^+^) = 1.4 * 10^8^ nm^3^ s^−1^ translates into a proton uptake rate of 14 s^−1^. Consequently, at steady state the 20 RC can reduce 125 QH2 per second which then allow the *bc*_1_ to pump 500 H^+^ s^−1^. Even under strong illumination this is only slightly more than the maximal turnover of the ATPase of 400 H^+^ s^−1^.

For the unbinding of the oxidized cytochrome *c*_2_ two experimental estimates of 270 s^−1^ [33] and 800 s^−1^ [34] exist which are both slower than our optimized value of 2200 s^−1^ indicating that the *c*_2_ are only loosely bound to the RCs. The corresponding binding rate of 9.2 * 10^5^ nm^3^ s^−1^ ensures that even when nearly all *c*_2_ are oxidized the supply with electrons from the reduced *c*_2_ does not become rate limiting.

In the steady state reconstruction the unbinding of the QH2 from the RC was estimated to take 25 ms [35] which made it the throughput-limiting process under steady state conditions, Here the optimized value of 87 s^−1^ is about twice as fast and the binding of the Q is even faster with an 80% reduced quinone pool.

**BC1**: For the *bc*_1_ dimers one of the optimization criteria was that the steady state throughput at vanishing ΔΦ is close to the experimentally determined value of about 75 *c*_2_ reduced per second [32]. Under such conditions the 10 *bc*_1_ dimers could consequently pump up to 1500 protons per second into the vesicle. For the *bc*_1_, the throughput-limiting reaction was found to be the unbinding of the oxidized Q from the Q_o_ site with a bc1::koff(Q@Qo) = 28 s^−1^. We also found that both the binding and the unbinding of the quinols is two to three times faster at the Q_i_ site than at the Q_o_ site. Additionally, most of the Q directly hop over from the Q_o_ to the Q_i_ site and only a few of them unbind from the *bc*_1_ back into the bulk. The same behavior was found for the QH2 generated at the Q_i_ site indicating that the direct transfer between the Q_i_ and the Q_o_ sites of the dimers is important for an efficient turnover as it effectively increases the local density of substrates. When any of these rates related to quinone dynamics at the Q_o_ and Q_i_ sites was scanned individually, the score changed only very little or not at all with the parameter value when the other rates were close to their optimal values. This occurs because, for example, Q can arrive at the Q_i_ site either from the Q pool in the membrane or from the nearby Q_o_ site of the other monomer. If either of these two paths is shut down due to a too low rate constant, the other path takes over and the *bc*_1_ continues to work. In the systemic multi-parameter optimization, however, such extreme settings are less favourable because they are more sensitive to small parameter changes and therefore effectively suppressed. This is why we obtained meaningful rates even for these correlated parameters.

As an initial estimate we had set the cytochrome *c*_2_ association and dissociation rates at the *bc*_1_ to the same values as at the RC, but the optimized rates are much faster. The binding of the *c*_2_ to the *bc*_1_ was about one order of magnitude faster than at the RC and the unbinding was found to be three times faster.

Our model explicitly includes the conformational change of the Rieske group between the *b* and the *c*_1_ domains associated with the electron transfer to the *c*_1_ heme. Here we found that the “forward” swing from the *b* to the *c*_1_ domain was about 30% faster than the corresponding motion back to the *b* domain. One can speculate that this is due to the proton release into the vesicle interior, which is gated by the Rieske domain, i.e., that when there are two protons waiting to be released they are pressing against the closed gate accelerating the opening conformational change.

**PR**: We found that some representation of protonatable residues (PR) must be present in the simulation. The optimal pK was about 5, but it could be varied between below 4 and 6.5 without any noticable differences in the time courses. Also their number was uncritical as long as it was above some 60 or 70 residues. This means that we considered on average a single protonatable group per protein. More protonatable residues would also mean that more protons can be pumped into the vesicle for the same *averaged* pH increase. Their charges, however, are not compensated for and the weight of ΔΦ_cap_ relative to ΔΦ_chem_ would increase further.

**ΔΦ**: Interestingly, the optimization found an effective electric capacitance close to the expected values, whereas the chemical contribution was found to be rather unimportant for the small vesicle with its protein-filled membrane. The small spread of good ΔΦ::U_0_ values emphasizes the relatively high importance of the charges for a faithful representation of ΔΦ, while in our simulations the chemical contribution from the proton density difference could have even be neglected completely.

### Relative Importances of the Experiments

**Figure 6:**
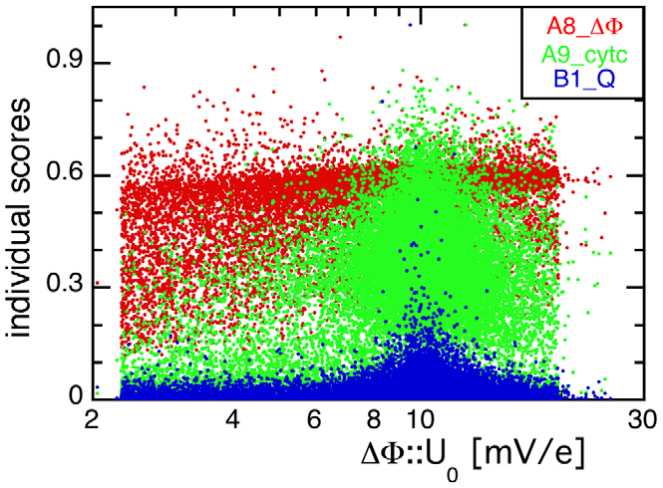
Different Experiments yield Different Sensitivities in the Parametrization. Projection of the individual scores of A8_ΔΦ (red points), A9_cytc (green points), and B1_Q (blue points) onto the value of the effective inverse capacitance of the vesicle ΔΦ::*U*_0_, illustrating that different scenarios may show different sensitivities with respect to a given parameter. The sensitivities for ΔΦ::*U*_0_ in these three experiments are 0.336 (A8_ΔΦ), 0.667 (A9_cytc), and 0.690 (B1_Q) from the respective 1000 best parameter sets.

As already mentioned above, experiments probing different setups and different kinetic regimes are sensitive to different parameters. This is illustrated in figure 6 which shows the projections of the individual scores from the three scenarios A8_ΔΦ, A9_cytc, and B1_Q onto the value of ΔΦ::U_0_ analogous to the distributions of the master score shown in figure 4. The individual score for the quasi-steady state scenario A8_ΔΦ is nearly independent of the electric capacitance with only a slight preference for larger values (red points in figure 6). According to this individual score, the respective sensitivity *P*_min_/*P*_max_ equals 0.336 from the 1000 best parameter values. Such a broad distribution with a low *P*_min_/*P*_max_ ratio denotes that this experiment is less important for the overall parameter determination. This correlates with the fact that buffering capacities do not play an important role under steady state conditions. However, a constant illumination of nine seconds length is not yet a true steady state. Indeed, the complementing measurement A9_cytc, which has the same illumination profile, shows a preference for values of ΔΦ::U_0_≈10 mV/e with a higher sensitivity of 0.667. In the third example, B1_Q, good scores are only obtained with ΔΦ::U_0_ close to its optimal value. This comes somewhat unexpected because this experiment with its wide-spaced flashes and the focus on the Q_B_ dynamics does not seem to probe the proton buffering capacity of the complete vesicle. Actually, with a value of 0.690, the sensitivity from the best 1000 parameter values is only slightly larger than for the apparently broader distribution from A9_cytc.

For each of the scenarios we determined an “importance score” by summing up the sensitivities *P*_min_/*P*_max_ of all relevant parameters. The resulting scores, listed in table 2, show that A9_cytc, B6_cytc, and A7_ΔΦ were most important for the parameter optimization. Interestingly, the quasi-steady state experiment A9_cytc was even more important than the many-flash scenario B6_cytc with its fast transients. In comparison, their respective companion experiments A8_ΔΦ and B6_P contribute far less to the parameter determination. On the other end of the spectrum is A7_cytc with its very fast single flash kinetics. B1_Q and BC1 both have low scores because they only probe a subset of the proteins of the complete vesicle. However, summing up their non-overlapping parameter coverages gives a rather good score of 9.3 indicating that both experiments performed better within their respective parameter subsets than their importance scores would indicate.

**Table 2:** Importance scores and correlation coefficients between the master score and the respective individual scores of the experimental scenarios denoting the relative importance of each of the experiments for the parameter value optimization.

experiment	A7_cytc	A7_ΔΦ	A8_ΔΦ	A9_cytc	B1_Q	B6_P	B6_cytc	BC1
importance score	4.4	7.7	5.8	9.7	3.8	5.2	8.9	5.5
correlation	0.09	0.44	0.22	0.38	0.83	0.17	0.31	0.41

The importance scores are determined as the sums of the sensitivities of all relevant parameters against the individual scores (see table S2 for all the individual values). The correlation coefficients are obtained from a linear fit of the master score against the respective individual score.

### Difficulties in the Modelling Process

One of the major uncertainties when setting up the simulations was the exact time course of the illumination during the single- and multiple-flash scenarios. We used a profile where the light was switched on and off instantaneously. In reality, however, the brightness of a flash bulb increases and then decreases again continuously with possibly two different time constants. This may explain why in the simulated flash experiments (A7_cytc, B6_P, and B6_cytc) the oxidation states of the cytochrome *c* and of the special pair decay faster than in the experiments. These simulations are also sensitive to the peak value of the intensity which we could not determine from the description of the experimental setup. The best we could do was to estimate the intensity based on the verbal description as “single turnover flashes”. The same uncertainty applies to the steady state scenarios A8_ΔΦ and A9_cytc. Here the intensity was described as “saturating” which is true for any intensity above some 20 W/m^2^. However, the steady state is reached faster with a higher light intensity and this would have been a way to tune the results of A8_ΔΦ and A9_cytc.

Starting from the optimized parameter values of table I, we also performed scans where only a single parameter was varied. Probing LHC::σ showed that its narrow distribution was essentially due to B1_Q alone. All the other individual scores only showed minor variations when LHC::σ was changed by up to an order of magnitude in either direction (see figure S4). To achieve semiquinone oscillations in B1_Q, the probability for an electron transfer in the RC during a flash has to be close to one. It is determined by the product of light intensity and absorption cross section which are therefore probed simultaneously. Any error in the description of the light intensity during the flash results in a wrong estimate for LHC::σ. On the other hand, our value for LHC::σ is close to the previously estimated value of 4.9 m^2^ W^−1^ s^−1^ [13] indicating that our guesses for the flash intensities worked quite well.

Another source of deviations is that we only simulated a single chromatophore, i.e., the parameters and simulation conditions were exactly the same for all repeated runs, whereas the experiments were performed on a collection of similar but non-identical chromatophores. To correctly account for the variations found in a real bacterium, one could run combined simulations with different chromatophore configurations, i.e., vesicles with different stoichiometries, sizes, and possibly leaks, using the same values for the individual rate constants. Along this line, one could in the future also investigate the effects of variations of the rate constants from protein to protein.

Closely related is the issue that the rate constants were optimized for a fixed vesicle size and stoichiometry. The determination of *N*_bc1_ had shown that varying the relative stoichiometries can easily change the dynamic behavior of the model. On the other hand, when the complete system is scaled such that all copy numbers are, for example, doubled, and the pools sizes are rescaled by the same factor, then the dynamic behavior remains unchanged. When only the vesicle diameter is increased then the surface area grows quadratically with the diameter and the inner volume with the third power. Consequently, the copy numbers of the transmembrane proteins and the volume of the quinone pool would increase with the same ratio, whereas the volume of the cytochrome *c* and proton pools grows faster. To recover the optimized binding probabilities, which are determined from the product of the association rate and the number of metabolites per pool volume, the association rates for protons and cytochrome *c* have to be rescaled accordingly. The rates for dissociation or internal conversion reactions remain unchanged because they do not depend on the respective pool volumes.

Our model is also incomplete in a kinetic sense as this study is focussed on the dynamic behavior of the photosynthetic apparatus in anaerobic cells of *Rb. sphaeroides* on time-scales from milliseconds up to a few seconds. Processes that only occur on longer time-scales were not included in the model such as regulatory effects on gene expression due to adaption to changes in the environmental conditions as, e.g., from aerobic to anaerobic growth. Also not considered were charge or redox relaxations in response to external buffers. Such exchange reactions have to be much slower than the turnover of the photosynthetic apparatus in order not to degrade its performance by acting like a shortcut to proton or redox gradients. However, an externally set redox poise in the experiments was considered in the simulations via appropriate initial redox states of the cytochrome *c*_2_ and quinone pools. Scenario A8_ΔΦ allows to derive an estimate for the time scales of such redox or charge relaxation processes. In this experiment ΔΦ_cap_ overshoots and relaxes again within less than one minute. For comparison, the ATPase can phosphorylate about 6000 ADP molecules during this time. Unfortunately, the description of the experiment does not state whether this is a reversible or an irreversible relaxation process.

### Conclusions

We have shown for the well understood model system of the photosynthetic apparatus of the purple bacterium *Rhodobacter sphaeroides* that the kinetic parameters for a dynamic model of a metabolic system can be reliably determined by simultaneously fitting the results from stochastic dynamics simulations against a number of time-dependent experiments. After determining an optimal set of kinetic and biophysical parameters for the chosen experiments with an evolutionary algorithm, our molecular stochastic simulation model can now reproduce the observed dynamics over a wide kinetic range from millisecond long single-flash experiments up to quasi steady state conditions. For this, we included 25 of the 44 parameters of the model in the optimization procedure. The few remaining deviations can be traced back to simplifications of the model and missing information about the experiments. With this approach we have thus successfully combined a detailed microscopic model built from molecular biological data and a systems biological parameter optimization by comparing the completely assembled system to macroscopic experimental observations. To be able to reproduce the time dependent experiments we first had to amend our model which had worked well for steady state scenarios before. The two main changes with respect to our previous model were the treatment of the transmembrane potential with the explicit contribution of the charges of the protons and the much more detailed model of the *bc*_1_ dimer which now allows to probe its internal states, too. After the systemic optimization we could then interpret the obtained optimal parameter values in the molecular realm. Thus, the two approaches were connected in both directions: molecular data lead to a systemic description and the behavior of the complete system identified important components of the molecular description.

Remarkably, only about one third of the stoichiometries, rate constants, and parameters of the model, which are all related to the kinetic behavior of the chromatophores, have sofar been determined experimentally. For the others only estimates were available. It was therefore not clear *a priori* whether a parameter determination would actually succeed in simultaneously narrowing down such a large number of parameters so that a reasonable agreement between the experiments and the simulation could be achieved. In fact, the evolutionary optimization found reasonable parameter sets already within the first ten iterations. During the next 30 iterations, the quality of these parameter sets was further increased. The evolutionary optimization not only gives the best parameter values but also, via the spread of the “good” solutions, allows to judge the importance of each parameter and of each experimental scenario used for the comparison. In the largest optimization run 54400 parameter sets were tested, optimizing 25 parameters simultaneously. The same number of data points on a 25 dimensional grid would mean less than two grid points per dimension. Consequently, a systematic scan for appropriate parameter values over intervals spanning several orders of magnitude each would have been absolutely impossible. On the other hand, the convergence of the scores when optimizing 25 parameters was not much slower than the convergence of the next smaller runs with 15 and 12 variable parameters, respectively. This indicates that optimization runs appear feasible using this approach where up to 25 parameters are optimized simultaneously. We suggest that the underlying fitness landscape has a funnel shape such as for protein folding which may explain why the stochastic dynamics of the evolutionary optimization successfully recovered the “native state” in such a high-dimensional space. The most efficient strategy for systems where less prior knowledge is available than for the very well-studied bacterial photosynthesis is therefore to first search within really wide parameter ranges and then iteratively confine the search space to those parameter regions where good scores were obtained. An evolutionary optimization algorithm quickly finds “good” solutions but the convergence is relatively slow. Therefore, this iterative refinement of the parameter ranges is more efficient than trying to obtain converged results from a single optimization step.

In total we spent more than 10 single-CPU-years of computing time on the optimizations presented here. For such a simple system, this may sound like a prohibitive amount of resources. However, with the experience from this project subsequent optimizations can be performed much more efficiently. For example, we used a very conservative short time step and relatively long initial equilibration phases. The last optimizations that we performed during this project could already be performed about three times faster than the first tests. Also the simulation code can be further optimized or parallelized to make efficient use of GPUs. Consequently, at least ten times larger systems can be optimized already now and even larger ones in the near future. However, for these larger and more interesting systems the bottleneck will not be the computational resources but the amount of available experimental data which is required to parametrize the many kinetic constants. Therefore it might actually be a more promising approach to parametrize the enzymes individually before they are plugged together. Such optimizations can already now be performed overnight on an average desktop computer.

The good agreement is remarkable also when considering that the experiments used as reference were not designed for a quantitative analysis of the photosynthetic apparatus but to elucidate the role of a specific protein, PufX. They were therefore not all performed under the very same external conditions. Also, some basic details such as the light intensities inside the samples or the actual intensity profiles of the flashes, which are crucial for the simulations, were not reported because they were not relevant for the questions asked. Based on the current results we expect that an even more reliable parameter determination and a more stringent verification of the simulation model could be performed with a set of experiments specifically designed for reproducible, quantitative measurements. Some of these experiments would be routine tests like applying the same dynamic illumination profiles under varying external redox or pH conditions. For the optimization presented here, only four different observables were measured, but over a wide range of timescales. This then allowed to reliably determine even parameters that are some reactions steps away from the measured quantities. It is therefore crucial that the set of experiments covers a wide range of the kinetic regime accessible to the system under consideration. Based on the experiences of this study, we suggest that one needs about *N*/5 independent kinetic experiments to be able to narrow the room of solutions for a cellular system with *N* reactions to a single basin of attraction. More work on other systems is certainly required to substantiate this claim.

During the optimization we did not find any unexpected parameter values, which would have indicated that our model was partly incorrect or not complete enough. For the wellknown bacterial photosynthesis we could set up the proteins from agreed upon biochemical models, but for less thoroughly studied systems the implementation might actually require the decision which of the available models to choose. Then, such a systemic parameterization can be also used to test which of the presented models behave correctly and potentially even to figure out why a certain model fails.

The obtained fully parametrized model gave important biological insights about the complete electron transport chain, especially about the central cytochrome *bc*_1_ complex and the quinones. Most importantly, we obtained values for the initially missing two thirds of the kinetic parameters of the model. For the *bc*_1_ complex we found that the quinones and quinols are exchanged preferentially between the two halves of the dimer in a local micro-environment with more favorable effective quinone oxidation states than in the bulk. However, the model of the bacterial photosynthetic apparatus as presented here is by no means complete. The design citeria for the individual proteins was to be able to reproduce the selected experiments which focus on the enzymatic functions of the proteins. Explicitly excluded are therefore all regulatory processes, while some of the very fast exciton and electron kintics inside the proteins could be modeled sufficiently well with effective reactions. When new experiments are added to the current set it is straightforward to update the current protein “templates” to the then required level of detail and thus to test these improved models for different observables and on a wider range of timescales.

Here we used the well established photosynthetic apparatus of *Rb. sphaeroides* to show that molecular data can be compiled with our pools-and-proteins approach to obtain consistent systemic answers. In this case of a well understood biological system wrong or inappropriate results can be identified *a posteriori*. However, the success indicates that the methodology can also be applied to other more complex or less well understood cellular subsystems. The next logical step at a higher level of complexity would be the bioenergetic processes of an entire bacterium or mitochondrium. Also signalling processes are good candidates for such a bottom-up molecular stochastic description together with the systemic evolutionary parametrization. In the case of the respiratory system, some of the proteins, namely the cytochrome *bc*_1_ complex and the ATPase, can be taken unaltered from the current model of photosynthesis together with their optimized parameters. Modeling and parametrization of other biological systems can thus be started from the already known proteins. By this, a library of protein models can be built up, with which finally the complete metabolism of an entire cell could be simulated. The resulting models at the molecular scale can also be used to test or verify under which conditions simplifications may be applied to reduce the complexity of larger models without changing their dynamic response. Correspondingly, we expect exciting progress in this area in the near future.

The molecular stochastic simulation framework with all currently implemented proteins can be obtained from the authors at http://service.bioinformatik.uni-saarland.de/vesimulus free of charge for academic use.

## Methods

### Evolutionary Parameter Optimization

**Figure 7:**
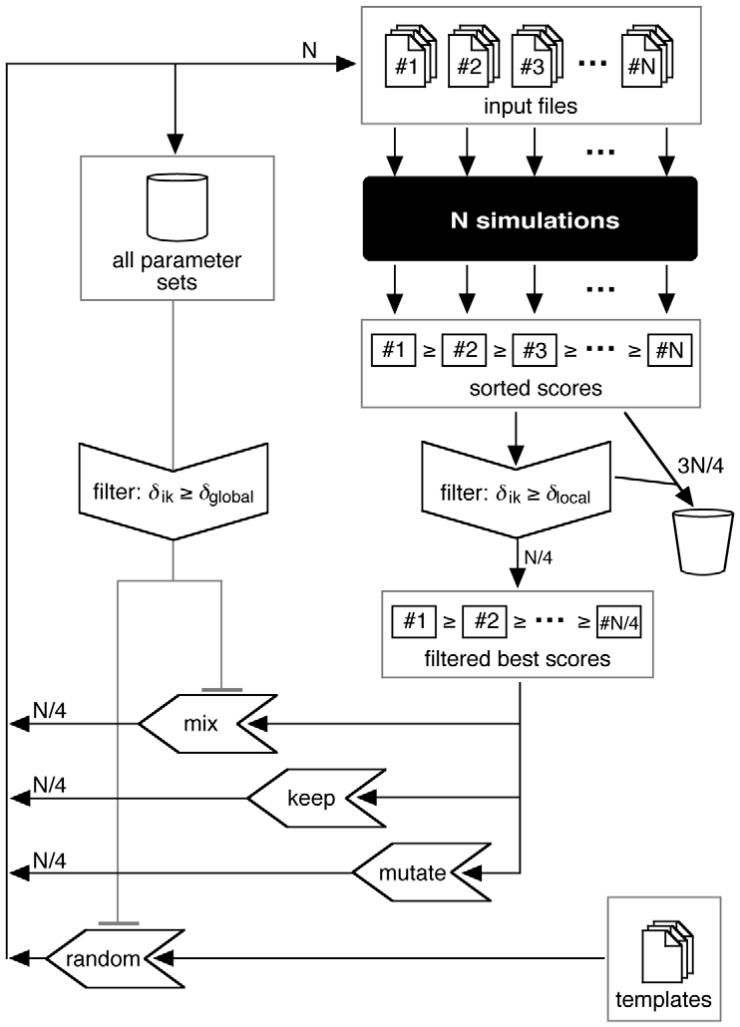
Sketch of the Evolutionary Algorithm Used to Optimize the Kinetic Parameters. The optimization algorithm and its modifications are described in detail in the main text.

The automated parameter optimization was performed with an evolutionary algorithm as introduced by Rechenberg [36] which is based on the biological ideas of repeated selection and mutation among a set of solutions, called a generation. An evolutionary optimization strategy can deal well with a high-dimensional search, for which no derivatives of the objective function are available and even multiple good solutions of comparable quality may exist. The algorithm is sketched in figure 7. It starts from a randomly initialized generation of *N* parameter sets. For this, the parameter values were distributed equally on a logarithmic scale within *a priori* chosen boundaries. From this initial generation of parameter sets, a set of input files for the simulation engine was generated from the template input files. Each of the templates describes one simulation setup corresponding to one of the experiments. Then, for each individual parameter set the set of stochastic simulations was run and the master score was determined as explained in the results section. For the next iteration, the *N*/4 parameter sets of the current generation with the best scores were retained unchanged (operation “keep” in figure 7). These simulations were repeated once more to ensure that their parameter sets did not score so well by chance due to stochastic fluctuations. Another *N*/4 individuals were generated as modified (mutated) copies of the best individuals by randomly changing each of the parameters considered in the optimization within a range of ±35%. The next *N*/4 new parameter sets were “cross-bred” by randomly selecting two sets from the best and taking the arithmetic average of their parameters (operation “mix”). The remaining *N*/4 slots were filled up with randomly initialized parameter sets. Again, a set of input files for the simulations was generated from the templates, and the parameter sets were scored by running and analyzing the respective simulations. This mutation-scoring-selection process was iterated until the scores converged. Limits for the parameter values were imposed only for the random initialization, but not for the subsequent mutations where they were allowed to take arbitrary values. However, in the optimizations performed here, the optimal values for the parameters were always found within the *a priori* estimated intervals.

To increase the convergence of the evolutionary optimization and to prevent that a complete generation gets stuck in a single local optimum, we extended the standard algorithm by two distance constraints between the parameter sets. For the first global constraint, all parameter sets that had been considered so far, including the ones in the latest generation, were saved in a global history. Each newly generated parameter set, be it from the averaging or from the random initialization, then had to differ by a predefined distance δ_global_ from any of these already considered parameter sets. This distance δ was calculated as a normalized Euclidian distance between the vectors of parameter values **p**_1_ and **p**_2_:
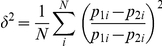
If a newly created parameter set was within δ_global_ of any of the previously scored parameter sets, it was discarded and replaced by a new randomly chosen parameter set—which was again tested. This approach avoided rerunning the relatively expensive simulations for a region of the parameter space that had already been examined previously. For the mutations, no such distance criterion was applied to allow for arbitrarily small improvements.

A second local distance constraint was applied when selecting the best *N*/4 from a generation. Starting from the parameter set with the best score, the next best set was only considered when its parameter vector was further away than δ_local_ from any of the already selected sets. If not, it was skipped and the next best parameter set was tested until enough good parameter sets were collected. With this criterion we could avoid that all parameter sets chosen for the next iteration were located in the same local optimum. Obviously, δ_local_ should be chosen at least as large as δ_global_. The benefits of using these two criteria were confirmed by testing them with specially crafted test cases which contained multiple maxima of different widths and heights. Currently, the minimal distances between two parameter sets are independent of the actual parameter values or the scores achieved in a certain region of the parameter space. In a more sophisticated implementation, they could be related to the scores of the parameter sets and increase the minimal distance for low scores, while allowing for smaller distances where the scores are good. Thus, the regions of parameter space where the experiments cannot be reproduced at all would be sampled more sparsely than the interesting regions.

Because all parameter sets of a given generation are scored independently, the parameter optimization with an evolutionary algorithm can be conveniently parallelized by running the simulations for different parameter sets on different nodes of a compute cluster.

### Parameter Search Ranges

For the random initialization of new parameter sets we had to specify a range for each parameter to be optimized. These intervals should be wide enough so that the optimal parameter value is inside the interval. On the other hand, the convergence of the optimization is faster when a smaller interval is used. For the well known bacterial photosynthesis we estimated the search ranges from various sources of information. For some of the parameters like the dissociation constant of the *c*_2_ from the RC experimental values were available around which the interval could be centered. Often, a lower limit for a parameter could be estimated from the steady state throughput of the respective protein. Alternatively, we ran tests where a single parameter was varied manually to get an estimate of the required range. With this first set of intervals the two main parameter optimization runs were performed. These ranges are listed in table S3.

To validate that the chosen intervals, which for some parameters spanned only one order of magnitude, were indeed wide enough, we also ran optimizations with very wide intervals for the initial values which spanned four to six orders of magnitude (see table S3). With these wide intervals optimization runs were performed where 25 parameters were optimized simultaneously. Due to the higher dimensionality and the incresed size of the parameter initialization intervals, the size of the multidimensional search space increased tremendously and the resulting score distributions could not be analyzed reliably anymore. However, by looking at the projected score distributions we were able to estimate the most important regions for each of the parameters. These ranges, which are also given in table S3, confirmed our initially chosen search ranges for the two main optimization runs. For some of the parameters we even found the same well defined optimal values as with the initial smaller ranges (see figure S5).

## Supporting Information

Figure S1 Determining the Number of *bc*_1_ Complexes: Evolution of the master score.
(0.07 MB PDF)

Figure S2 Determining the Number of *bc*_1_ Complexes: Average Master Score vs. the number of *bc*_1_ complexes *N*_bc1_.
(0.09 MB PDF)

Figure S3 Evolution of the Scores for Different Subgroups.
(0.10 MB PDF)

Figure S4 Scores from Some Experiments During the Scan of One Parameter.
(0.09 MB PDF)

Figure S5 Comparison of a Score Distribution Obtained from a Main Parameterization Run and from a Wide Range Verification Run.
(0.98 MB PDF)

Table S1 Model Parameters Not Included in the Optimization Process.
(0.08 MB PDF)

Table S2 Sensitivities of the Experimental Scenarios.
(0.08 MB PDF)

Table S3 Search Ranges for the Parameter Optimization.
(0.08 MB PDF)

Text S1 Fit Functions to the Experimental Time Traces.
(0.24 MB PDF)
