# Supplementary material for: Bridging the Gap: Linking Molecular Simulations and Systemic Descriptions of Cellular Compartments
Source: PLoS One. 2010 Nov 22;5(11):e14070. doi: 10.1371/journal.pone.0014070 (PMC2989909; doi:10.1371/journal.pone.0014070)
Supplement: Figure S1 — Determining the Number of bc 1 Complexes: Evolution of the master score. (0.07 MB PDF) [file pone.0014070.s001.pdf]

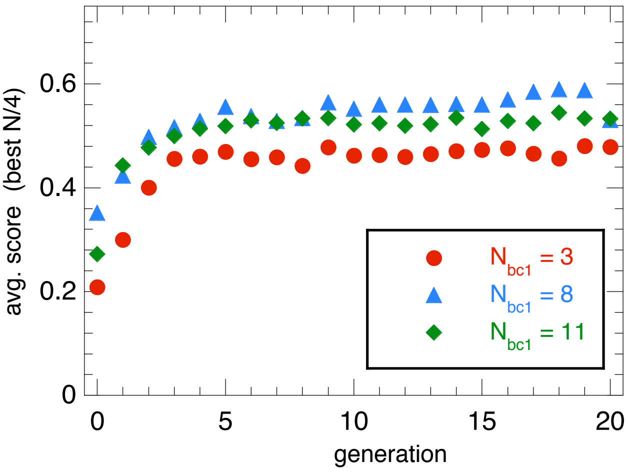

Figure S1: Determining the Number of bc1 Complexes: Evolution of the Master Score

Convergence of the average master scores of the best 100 parameter sets during three optimization runs with different numbers of bc_1 complexes N_{bc1}. These optimizations used $N = 40$ individuals per generation.
