# Supplementary material for: Bridging the Gap: Linking Molecular Simulations and Systemic Descriptions of Cellular Compartments
Source: PLoS One. 2010 Nov 22;5(11):e14070. doi: 10.1371/journal.pone.0014070 (PMC2989909; doi:10.1371/journal.pone.0014070)
Supplement: Figure S2 — Determining the Number of bc 1 Complexes: Average Master Score vs. the number of bc 1 complexes N bc1. (0.09 MB PDF) [file pone.0014070.s002.pdf]

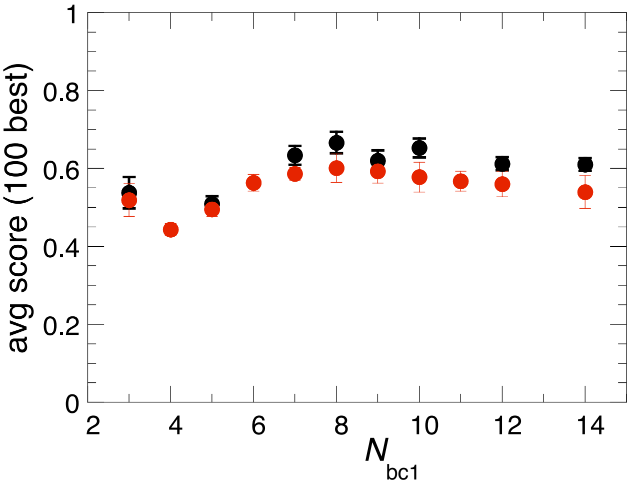

Figure S2: Determining the Number of bc_1 Complexes: Average Master Score

Average master score of the best 100 (out of a total of 840) parameter sets vs. the number of bc_1 complexes N_{bc1} for the single flash experiment used to determine N_{bc1}. This value of N_{bc1} was later employed for the optimization of the rate constants. The black points show the result from the first set of optimizations that considered $\Delta\Phi$::ε_M, $\Delta\Phi$::$\Delta\Phi_0$, and bc_1::Φ_0. In the second set (red points), PR::pK was included, too.
