# Supplementary material for: Bridging the Gap: Linking Molecular Simulations and Systemic Descriptions of Cellular Compartments
Source: PLoS One. 2010 Nov 22;5(11):e14070. doi: 10.1371/journal.pone.0014070 (PMC2989909; doi:10.1371/journal.pone.0014070)
Supplement: Figure S3 — Evolution of the Scores for Different Subgroups. (0.10 MB PDF) [file pone.0014070.s003.pdf]

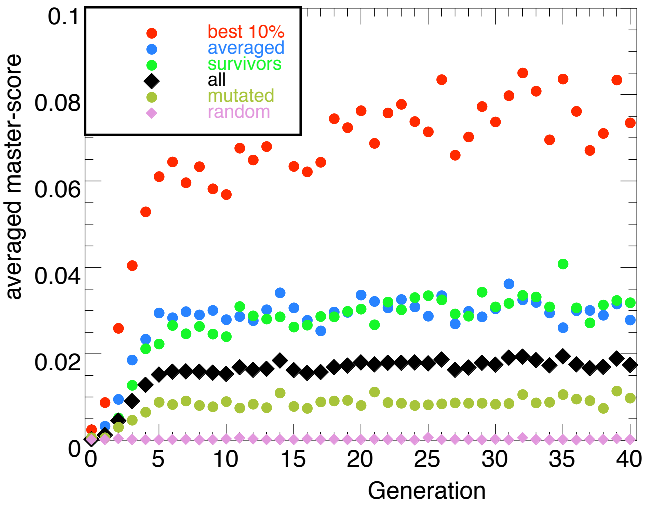

Figure S3: Evolution of the Scores for Different Subgroups

Shown is the convergence of the average scores computed for different subgroups of the population during the largest optimization run considering 17 parameters simultaneously over 40 iterations of 800 individuals each. The new individuals of each generation, which were created with random parameter values, constantly had a rather low score (data points with label "random"). The score averaged over the whole generation (label "all") first increased and then saturated after about ten generations. The group labelled "survivors" are the best 25% of the previous generation. Consequently, the average score of this group was higher than the total average. The same applied to the "averaged" subgroup, which consisted of individuals with parameter values that are the averages from two individuals from the best 25%. However, as the two individuals for this "mating" process were chosen randomly from the best, the score for the "averaged" subgroup exhibited slightly larger fluctuations than the "survivors". Both these groups had the same convergence as the average from the complete generation. Interestingly, the score of the "mutated" subgroup was lower than the overall average. The reason for this is that in such a high-dimensional parameter space the chances to move away from an already good parameter set are much higher than to improve on its quality. The most interesting subgroup are the "best 10%" of each generation. Their average score was more than twice as large than that of the next best group. An onset of saturation can be seen from generation 30 on. The evolution of the scores as shown here is typical for the optimizations performed.
