# Supplementary material for: Bridging the Gap: Linking Molecular Simulations and Systemic Descriptions of Cellular Compartments
Source: PLoS One. 2010 Nov 22;5(11):e14070. doi: 10.1371/journal.pone.0014070 (PMC2989909; doi:10.1371/journal.pone.0014070)
Supplement: Figure S4 — Scores from Some Experiments During the Scan of One Parameter. (0.09 MB PDF) [file pone.0014070.s004.pdf]

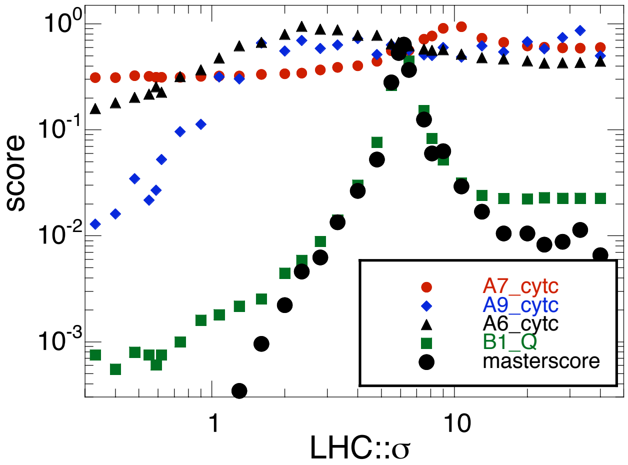

Figure S4: Scores from Some Experiments During the Scan of One Parameter

Masterscore and individual scores from four of the experiments obtained from a scan of LHC::σ starting from the optimal set of parameters. All scores were rescaled to coincide at the maximum value of the masterscore. The other individual scores, which are not shown, have an even weaker dependence on LHC::σ. One can see that the optimal parameter value is determined mainly by the scenario B1_Q, whereas A7_cytc would favor a larger value and A6_cytc and A9_cytc would perform better with a smaller value of LHC::σ.
