# Supplementary material for: Bridging the Gap: Linking Molecular Simulations and Systemic Descriptions of Cellular Compartments
Source: PLoS One. 2010 Nov 22;5(11):e14070. doi: 10.1371/journal.pone.0014070 (PMC2989909; doi:10.1371/journal.pone.0014070)
Supplement: Figure S5 — Comparison of a Score Distribution Obtained from a Main Parameterization Run and from a Wide Range Verification Run. (0.98 MB PDF) [file pone.0014070.s005.pdf]

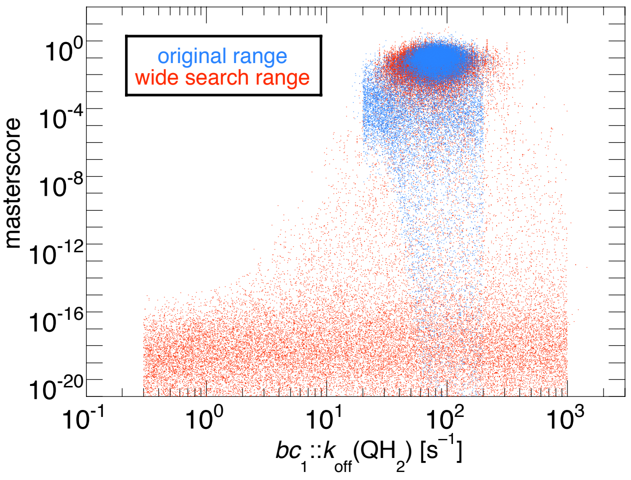

Figure S5: Comparison of a Score Distribution Obtained from a Main Parameterization Run and from a Wide Range Verification Run

In the main optimization runs the ranges for the random initialization of the parameter values were chosen based on biochemical or steady state estimates in order to get good statistics for the optimization. The initially estimated ranges where then verified by running additional optimizations with wide ranges for the parameter initialization which then spanned three to six orders of magnitude for each parameter. This figure illustrates on the example of the parameter bc_1::$k_{\mathrm{off}}(QH_2)$ how the masterscore falls off fast beyond the initially chosen interesting parameter range. From the wide range scan one can see that the parameter should be larger than 30 s^{-1} with a—visually estimated—optimal value of about 70 s^{-1}. A complete list of search ranges and parameter estimated is given in table S3.
