# Supplementary material for: Bridging the Gap: Linking Molecular Simulations and Systemic Descriptions of Cellular Compartments
Source: PLoS One. 2010 Nov 22;5(11):e14070. doi: 10.1371/journal.pone.0014070 (PMC2989909; doi:10.1371/journal.pone.0014070)
Supplement: Table S1 — Model Parameters Not Included in the Optimization Process. (0.08 MB PDF) [file pone.0014070.s006.pdf]

Parameter	Value	Description
bc_1::k_{on}(H^+_{out})	10^{10} nm^3 s^{-1}	rate for proton uptake from the cytoplasm by bc_1
bc_1::k_{tr}(e:Q_o=>FeS)	2.3 * 10^3 s^{-1}	rate for electron transfer from Q_o to FeS
bc_1::k_{tr}(e:c_1=>c_2)	10^5 s^{-1}	electron transfer rate from c_1 to bound cytochrome c_2
bc_1::k_{tr}(e:Q_o=>b_L)	10^4 s^{-1}	electron transfer from Q_0 to b_L heme
bc_1::k_{tr}(e:b_L=>b_H)	10^4 s^{-1}	electron transfer from b_L to b_H heme
ΔΦ::V	2.65 * 10^4 nm^3	inner volume of the vesicle
ΔΦ::A	5.28 * 10^3 nm^2	membrane area (Q pool „volume“)
ΔΦ::C_{Hin}	1.0 e	effective charge of a free proton in the vesicle
ΔΦ::C_{Hm}	1.0 e	effective charge of a proton on the titratable groups
ΔΦ::C_{prot}	–1.0 e	effective charge of an e^- translocated through an RC
ΔΦ::C_{cred}	–0.5 e	effective charge of a reduced cytochrome c_2
ΔΦ::C_{cox}	0.5 e	effective charge of an oxidized cytochrome c_2
PR::N_p	80	number of titratable groups in the vesicle
PR::pK	5.0	pK of the titratable groups
N_{core}	10	number of dimeric core complexes (2 RC + 1 LHC)
N_{bc1}	10	number of cytochrome bc_1 complexes
N_{ATPase}	1	number of ATPases
N_{c2}	20	total number of cytochrome c_2
N_Q	200	total number of quinones

Table S1: Model Parameters Not Included in the Optimization Process

Parameters and stoichiometries that were not included in the optimization process with their (fixed) values. Effective charges of ±0.5 e were used for the cytochrome c_2 to achieve a change of the total charge of the cytochrome c_2 of ±1 e when a reduced c_2 is replaced by an oxidized one or vice versa. Note that N_{bc1} was determined prior to the main parameterization and is thus considered a fixed parameter for the chromatophore setup.
