# Supplementary material for: Bridging the Gap: Linking Molecular Simulations and Systemic Descriptions of Cellular Compartments
Source: PLoS One. 2010 Nov 22;5(11):e14070. doi: 10.1371/journal.pone.0014070 (PMC2989909; doi:10.1371/journal.pone.0014070)
Supplement: Table S2 — Sensitivities of the Experimental Scenarios. (0.08 MB PDF) [file pone.0014070.s007.pdf]

Parameter	A7 cytc	A7 ΔΦ	A8 ΔΦ	A9 cytc	B1 Q	B6 P	B6 cytc	BC1	avg.
LHC::σ	0.426	0.633	0.426	0.606	0.934	0.746	0.746	–	0.645
LHC::k_D(E)	0.048	0.216	0.052	0.155	0.254	0.032	0.219	–	0.139
RC::k_{on}(E)	0.006	0.005	0.071	0.244	0.049	0.023	0.088	–	0.069
RC::k_{on}(H^+)	0.006	0.005	0.658	0.893	0.148	0.154	0.340	–	0.315
RC::k_{on}(Q)	0.187	0.435	0.199	0.352	0.472	0.169	0.415	–	0.318
RC::k_{off}(QH2)	0.207	0.488	0.169	0.518	0.556	0.275	0.493	–	0.387
RC::k_{on}(c2red)	0.024	0.350	0.166	0.469	0.103	0.450	0.151	–	0.245
RC::k_{off}(c2ox)	0.216	0.485	0.182	0.337	0.440	0.149	0.488	–	0.328
bc_1::k_{on}(QH2@Q_o)	0.064	0.019	0.211	0.385	–	0.123	0.303	0.406	0.216
bc_1::k_{off}(Q@Q_o)	0.704	0.680	0.320	0.526	–	0.282	0.763	0.680	0.565
bc_1::k_{tr}(Q:Q_o=>Q_i)	0.200	0.426	0.213	0.373	–	0.171	0.398	0.422	0.315
bc_1::k_{on}(Q@Q_i)	0.040	0.037	0.201	0.413	–	0.123	0.204	0.392	0.201
bc_1::k_{off}(QH2@Q_i)	0.303	0.510	0.301	0.442	–	0.273	0.493	0.474	0.399
bc_1::k_{tr}(QH2:Q_i=>Q_o)	0.191	0.388	0.195	0.330	–	0.145	0.394	0.380	0.289
bc_1::k_{on}(c2ox)	0.156	0.339	0.134	0.270	–	0.106	0.394	0.310	0.244
bc_1::k_{off}(c2red)	0.022	0.020	0.119	0.233	–	0.067	0.102	0.198	0.109
bc_1::k_{off}(H^+@Q_o)	0.034	0.028	0.158	0.265	–	0.096	0.172	0.253	0.144
bc_1::k_{tr}(FeS:*b*=>*c*)	0.358	0.543	0.326	0.474	–	0.273	0.552	0.510	0.434
bc_1::k_{tr}(FeS:*c*=>*b*)	0.312	0.521	0.315	0.472	–	0.287	0.474	0.532	0.416
bc_1::k_{tr}(e:b_H=>Q_i)	0.144	0.361	0.145	0.300	–	0.126	0.286	0.346	0.244
bc_1::Φ_0	0.457	0.522	0.506	0.664	–	0.498	0.556	0.608	0.544
ΔΦ::ΔU_0	0.325	0.525	0.405	0.520	0.576	0.359	0.524	–	0.462
ΔΦ::$\Delta\Phi_0$	0.001	0.126	0.352	0.490	0.301	0.263	0.338	–	0.267
importance score	4.431	7.662	5.824	9.731	3.833	5.190	8.893	5.511	7.296

Table S2: Sensitivities of the Experimental Scenarios

This table lists the sensitivities P_{min}/P_{max} determined separately for each of the experiments from the respective individual scores. The values in the last column (labelled „avg.“), which are the averages of the individual sensitivities for each of the parameters, are mostly two to four times smaller than the respective sensitivities determined from the masterscore, which make use of all scenarios simultaneously (see table I). The last line lists the importance scores defined as the sum of the sensitivities for each of the experiments. A larger value denotes that this experiment is more important for determining the parameter values.
