# Supplementary material for: Bridging the Gap: Linking Molecular Simulations and Systemic Descriptions of Cellular Compartments
Source: PLoS One. 2010 Nov 22;5(11):e14070. doi: 10.1371/journal.pone.0014070 (PMC2989909; doi:10.1371/journal.pone.0014070)
Supplement: Table S3 — Search Ranges for the Parameter Optimization. (0.08 MB PDF) [file pone.0014070.s008.pdf]

Parameter [units]	Search Range in Run A1	Search Range in Run A2	Search Range in Run B6	Estimated Parameter Range from B6
LHC::σ [$m^2\ W^{-1}\ s^{-1}$]	3 … 20	—	0.1 … 100	5 … 32
LHC::N_0 [1]	—	0.1 … 10	—	
LHC::k_D(E) [s^{-1}]	10^2 … 10^5	—	1.0 … 3*10^4	≥ 150
RC::k_{on}(E) [s^{-1}]	—	10^5 … 10^9	2*10^2 … 2*10^9	≥ 10^4
RC::k_{on}(H^+) [$nm^3\ s^{-1}$]	—	10^6 … 10^{10}	2*10^6 … 10^{10}	(1.2 … 3.6) * 10^8
RC::k_{on}(Q) [$nm^2\ s^{-1}$]	5*10^3 … 3*10^5	—	50 … 2*10^6	≥ 5*10^3
RC::k_{off}(QH2) [s^{-1}]	10 … 200	—	0.1 … 10^4	≥ 12
RC::k_{on}(c2red) [$nm^3\ s^{-1}$]	—	4*10^4 … 10^8	10^3 … 5*10^7	≥ 2*10^5
RC::k_{off}(c2ox) [s^{-1}]	150 … 8*10^3	—	2 … 1.5*10^5	≥ 180
bc_1::k_{on}(QH2@Q_o) [$nm^2\ s^{-1}$]		10^2 … 10^5	30 … 3*10^6	≥ 1.6*10^3
bc_1::k_{off}(Q@Q_o) [s^{-1}]	15 … 150	—	1 … 250	≥ 15
bc_1::k_{tr}(Q:Q_o=>Q_i) [s^{-1}]	10^3 … 2.5*10^4	—	0.5 … 5*10^4	*
bc_1::k_{on}(Q@Q_i) [$nm^2\ s^{-1}$]	—	3*10^4 … 10^7	10 … 10^5	*
bc_1::k_{off}(QH2@Q_i) [s^{-1}]	20 … 200	—	0.3 … 10^3	≥ 30
bc_1::k_{tr}(QH2:Q_i=>Q_o) [s^{-1}]	8*10^2 … 2.5*10^4	—	0.5 … 5*10^4	*
bc_1::k_{on}(c2ox) [$nm^3\ s^{-1}$]	5*10^5 … 10^8	—	5*10^3 … 10^8	≥ 2*10^5
bc_1::k_{off}(c2red) [s^{-1}]	—	10^2 … 10^5	0.5 … 5*10^5	≥ 22
bc_1::k_{off}(H^+@Q_o) [s^{-1}]	—	10^3 … 5*10^5	10 … 5*10^4	≥ 110
bc_1::k_{tr}(e:Q_o=>FeS) [s^{-1}]	—	—	1 … 10^4	≥ 46
bc_1::k_{tr}(FeS:b=>c) [s^{-1}]	10^2 … 3*10^4	—	5 … 6*10^5	≥ 60
bc_1::k_{tr}(FeS:c=>b) [s^{-1}]	10^2 … 3*10^4	—	1 … 3*10^5	≥35
bc_1::k_{tr}(e:b_H=>Q_i) [s^{-1}]	3*10^2 … 5*10^4	—	1 … 10^5	>12
bc_1::Φ_0	60 … 250	60 … 250	1 … 5*10^4	46 … 3000
ΔΦ::ΔU_0	3 … 30	3 … 30	10^{-2} … 10^3	1.4 … 40
ΔΦ::ΔΦ_0	—	2 … 75	10^{-2} … 10^3	*
#Gen/#Ind.	40/800	40/800	34/1600	

Table S3: Search Ranges for the Parameter Optimization

This table gives the parameter ranges that were used for the random initialization of new parameter sets during the two main parameterization runs A1 and A2 and in the largest of the subsequent test runs (B6). In B6 25 parameters were optimized simultaneously compared to the 17 and 12 parameters in A1 and A2, respectively, with ranges that were wider by orders of magnitudes. From the results, which had much weaker statistics, we could not obtain converged parameter values, but from the distributions of the masterscore vs. the respective paraeter values we estimated in which ranges the parameters should be (also see figure S5). These estimates are given in the last column. Here, an

asterisk denotes that no clear range estimate could be read from a rather flat score distribution. These estimates confirm that that the initially chosen ranges indeed included the optimal parameter values. The last line lists the numbers of generations and individuals per generation for each of the runs. Runs A1 and A2 were performed with $\delta_{local} = 0.2$ and $\delta_{global} = 0.1$, while for the wide range run B6 a rather large value of 0.5 was used for both criteria, which broadened the resulting parameter distributions.
