# Supplementary material for: Bridging the Gap: Linking Molecular Simulations and Systemic Descriptions of Cellular Compartments
Source: PLoS One. 2010 Nov 22;5(11):e14070. doi: 10.1371/journal.pone.0014070 (PMC2989909; doi:10.1371/journal.pone.0014070)
Supplement: Text S1 — Fit Functions to the Experimental Time Traces. (0.24 MB PDF) [file pone.0014070.s009.pdf]

Fit Functions to the Experimental Time Traces

Before comparing the simulation results to the respective fit functions, the simulation results were scaled for best fit. Also the time axis was shifted such that the onset of the illumination profile starts at $t = 0$. The fit functions are therefore given in arbitrary units. The fit functions are shown in figure 5 of the main text together with the experimental data and the three best simulation results.

The fit to the oxidation state of the cytochrome c in **A6_cytc** was defined piecewise as

$$f(t_i < t \leq t_{i+1}) = c_i + d_i\,(1 - \exp[-k_i(t - t_i)]) + e_i(t - t_i)$$

with the coefficients given in the following table. The simulation results were multiplied by (–1) and scaled such that the first step had the same height as the fit function.

i	t_i [ms]	c_i	d_i	k_i [ms^{-1}]	e_i
1	30	6.3	0	0.0	0.0
2	60	10.7	0	0.0	0.0
3	90	10.7	1.4	0.3	0.0
4	120	12.2	0.8	0.2	0.0
5	150	13.0	0.4	0.2	0.0
6	180	13.4	2.0	0.009	0.0
7	770	18.64	0.0	0.0	2.0
8	1000	—	—	—	—

For **A7_cytc** the simulation result was scaled to have a maximum of the same height and was fitted against a sum of two exponentials

$$f(t) = c_0 + \sum_{i=1}^{2} c_i \exp[-k_i(t - t_i)]$$

with the following parameters:

i	t_i [ms]	c_i	k_i [ms^{-1}]
0	—	0.04	—
1	0.0	–0.17	0.13
2	0.1	2.5	0.28

A similar fit with three exponentials was used for **A7_ΔΦ**, too, but with $t_i = 0$ this time.

i	c_i	k_i [ms^{-1}]
0	0.61	—
1	–0.9	100
2	-1.03	0.48
3	1.33	0.012

For **A8_ΔΦ** the same fit function was used as for A6_cytc with the coefficients given in the following table. The simulation results were scaled such that the first step of ΔΦ directly after the flash at t = 0 has a value of 1.0.

i	t_i [s]	c_i	d_i	k_i [s^{-1}]	e_i
1	0.0	1	1.3	0.8	0.0
2	4.5	1	1.3	480	–0.07
3	8.95	0.48	1.5	8.0	0.0
4	11	—	—	—	—

The fit to **A9_cytc** was described by the same function as A8_ΔΦ. Here, the simulation was scaled to the same average value as the fit during the interval 2 s < t < 8 s. The coefficients of the fit function are given in the table below.

i	t_i [s]	c_i	d_i	k_i [s^{-1}]	e_i
1	0	0	–1.60	0.7	0.0
2	1.59	–1.078	–1.55	2.3	0.0
3	3.5	–1.231	0.0	—	0.04
4	9.05	–0.33	0.28	2.3	0.0
5	11	—	—	—	—

To ignore the flash-related spikes seen in the semiquinone oscillations of **B1_Q**, the simulation results were fitted against constant values d_i during the intervals $t_i + 0.4$ s < t < $t_i + 0.95$ s.

t_i [s]	0	1	2	3	4
d_i	0	1.38	0.41	2.15	0.55

The complex multi-spike measurements of **B6_P** were described by a sum of two fit functions $f_1(t) + f_2(t)$, whereby f_1 models the amplitude of the train of spikes and f_2 describes the individual spikes. The simulation results were normalized against the height of the first spike.

$$f_1(t) = 0.8 + 0.1 \exp[-25 \text{ ms}^{-1} \text{ t }] + \exp[0.1 \text{ ms}^{-1} \text{ t }]$$

$$f_2(t) = -c_i \, (1 - \exp[-k_i \, (t - t_i)]) - d_i \, (t - t_i)$$

i	t_i [ms]	c_i	k_i [ms^{-1}]	d_i
1	0.0	0.88	0.80	0.0
2	20	0.88	0.65	4.5
3	40	0.8	0.65	3.0
4	60	0.77	0.60	4.0
5	80	0.72	0.70	7.5
6	100	0.79	0.50	2.0
7	120	0.75	0.50	4.0
8	140	0.75	0.55	5.0
9	160	0.70	1.00	7.0
10	180	0.70	1.00	8.0
11	200	0.70	1.00	7.0
12	220	0.72	0.70	6.0
13	240	0.74	0.70	3.0
14	260	0.74	0.80	4.0
15	280	0.67	1.00	8.0
16	300	0.75	0.60	2.0
17	320	—	—	—

For the corresponding experiment **B6_cytc** we multiplied the simulation results with (–1) and used

$f_1(t) = 0.9 + 0.1 \exp[-35 \text{ ms}^{-1} t] + \exp[3 \text{ ms}^{-1} t]$

and the same $f_2(t)$ as for B6_P with the following parameters:

i	t_i [ms]	c_i	k_i [ms^{-1}]	d_i
1	0.0	0.82	0.70	3.0
2	20	0.70	0.60	8.0
3	40	0.70	0.70	5.0
4	60	0.65	0.70	8.0
5	80	0.65	0.75	8.0
6	100	0.63	0.70	9.0
7	120	0.63	0.75	8.0
8	140	0.63	0.60	9.0
9	160	0.64	1.0	7.0
10	180	0.62	0.85	10.0
11	200	0.65	0.75	7.0
12	220	0.64	0.65	9.0
13	240	0.65	0.55	8.0
14	260	0.64	0.70	9.0
15	280	0.63	0.78	8.0
16	300	0.70	0.50	15.0
17	320	—	—	—

To score the steady state throughput of the bc_1 complex in the scenario **BC1**, the simulation was run for 20 seconds with a single bc_1 and then the number of reduced cytochrome c_2, N_{c2}, was converted into the score as

$$s = \exp\left[-\frac{(N_{c2} - N_{avg})^2}{2\sigma^2}\right]$$

with $N_{avg} = 1500$ and $\sigma = 200$.
